# The Nedd4-2/Ndfip1 axis is a negative regulator of IgE-mediated mast cell activation

**DOI:** 10.1038/ncomms13198

**Published:** 2016-10-27

**Authors:** Kwok Ho Yip, Natasha Kolesnikoff, Nicholas Hauschild, Lisa Biggs, Angel F. Lopez, Stephen J. Galli, Sharad Kumar, Michele A. Grimbaldeston

**Affiliations:** 1 Centre for Cancer Biology, University of South Australia and SA Pathology, Adelaide, South Australia 5000, Australia; 2 School of Medicine, University of Adelaide, Adelaide, South Australia 5005, Australia; 3 Departments of Pathology and of Microbiology and Immunology, and the Sean N. Parker Center for Allergy and Asthma Research, Stanford University School of Medicine, Stanford, California 94305-5176, USA; 4 OMNI-Biomarker Development, Genentech Inc., South San Francisco, California 94080, USA

## Abstract

Cross-linkage of the high-affinity immunoglobulin E (IgE) receptor (FcɛRI) on mast cells by antigen ligation has a critical role in the pathology of IgE-dependent allergic disorders, such as anaphylaxis and asthma. Restraint of intracellular signal transduction pathways that promote release of mast cell-derived pro-inflammatory mediators is necessary to dampen activation and restore homoeostasis. Here we show that the ligase Nedd4-2 and the adaptor Ndfip1 (Nedd4 family interacting protein 1) limit the intensity and duration of IgE-FcɛRI-induced positive signal transduction by ubiquitinating phosphorylated Syk, a tyrosine kinase that is indispensable for downstream FcɛRI signalosome activity. Importantly, loss of Nedd4-2 or Ndfip1 in mast cells results in exacerbated and prolonged IgE-mediated cutaneous anaphylaxis *in vivo*. Our findings reveal an important negative regulatory function for Nedd4-2 and Ndfip1 in IgE-dependent mast cell activity.

## Abstract (web summary)

Aberrant activation of the IgE receptor on mast cells leads to allergic responses. Here, the authors identify an E3 ligase and adaptor protein that can reduce IgE signalling by targeting phosphorylated-Syk for degradation.

## Graphical abstract

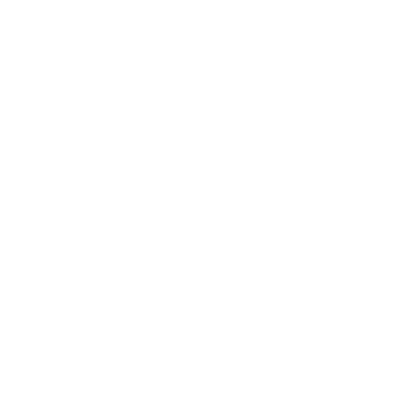

Although the roles of mast cells in inflammation can be complex (including evidence that they can negatively regulate inflammation in certain settings^1^,^2^,^3^,^4^,^5^), they are best known as efficient pro-inflammatory effector cells which can provoke strong immunoglobulin E (IgE)-mediated responses to allergens in sensitized individuals. Indeed, IgE-dependent elicitation of mast cell mediator production helps to drive the complex pathology of allergic disorders, such as atopic asthma, allergic rhinitis (hay fever), atopic dermatitis (eczema) and life-threatening anaphylaxis^6^,^7^. Therefore, a better understanding of the inherent regulatory mechanisms that can restrain the intricate signalosome and restore homoeostasis following IgE-mediated mast cell activation is essential for identifying new opportunities for therapeutic intervention.

Mast cells express on their surface the high-affinity IgE receptor FcɛRI and can be activated by multivalent antigen (Ag)-mediated aggregation of IgE-bound to the α-subunit of this receptor^7^,^8^. Activated mast cells degranulate within minutes of Ag exposure, releasing a diverse array of mediators, including proteases and vasoactive amines (for example, histamine), which characterize the early-phase response, or type I immediate hypersensitivity reaction. A later phase of the pro-inflammatory response reflects the *de novo* synthesis of lipid mediators (for example, prostaglandins and cysteinyl leukotrienes (LTD_4_, LTC_4_)), as well as cytokines and chemokines (for example, TNF, IL-6, IL-4, IL-13, MIP-1α (CCL3), MCP1 (CCL2))^6^,^7^.

At the molecular level, receptor oligomerization and subsequent engagement of the IgE-FcɛRI signalosome involves a complex series of phosphorylation events involving multiple activating Src family kinases, including Fgr (refs 9, 10), Fyn, Hck (ref. 11) and Lyn, upstream of Syk kinase^12^. Lyn can exert a positive role in activating mast cells through its phosphorylation of immunoreceptor tyrosine-based activation motifs (ITAMs) found within the cytoplasmic domains of the β chain and the two homodimer γ chains of FcɛRI^12^,^13^,^14^. In rapid succession, Syk kinase is activated in a process that is thought to involve Lyn^12^ and Fgr^9^, and is recruited to distinct binding sites in the γ subunit ITAM where it serves to amplify signal transduction. Key to this function and to its essential role in the calcium response, degranulation and cytokine production following FcɛRI engagement^13^, is the capacity of cytosolic Syk to interact with multiple signalling proteins. Syk is responsible for the phosphorylation of adapter molecules (for example, linker for activation of T cells; LAT1/2), required for assembly of the signal transduction machinery and downstream phosphorylation of pivotal mitogen-activated protein kinases (MAPKs) such as extracellular signal-regulated kinase (Erk1/2) as well as the transcription factors NF-κB and nuclear factor of activated T cells^15^.

FcɛRI engagement also promotes activation of several inhibitory receptors (for example, FcγRIIB, gp49B1, MAFA, PIR-B)^8^,^16^, as well as a range of negative regulators of intracellular signalling in the network (for example, RabGEF1 (ref. 17), SHIP (ref. 16), the protein tyrosine phosphatases SHP1 and SHP2 (ref. 12), and Lyn, which can exert positive or negative regulation depending on the intensity of the stimuli^14^). These mechanisms of negative regulation serve to counteract positive signalling and thereby determine the rate and extent of mast cell responses. A major, yet less understood, mechanism by which mast cells can negatively regulate their function is via ubiquitination. E3 ubiquitin ligases are responsible for the attachment of ubiquitin chains to select target proteins, a modification that can prompt endocytosis of cell surface receptors and initiate proteasomal or lysosomal degradation of signalling proteins^17^,^18^.

In this study, we identify a function in mast cells of the ubiquitin ligase Nedd4-2 (also known as Nedd4l (Neural precursor cell-expressed developmentally downregulated gene 4-like)), a member of the Nedd4 E3 family, as an important negative regulator of IgE-FcɛRI signalling and pro-inflammatory mediator release. Nedd4-2 contains an N-terminal C2 (Ca^2+^ dependent lipid binding) domain, 4 WW domains that enable direct protein–protein interaction and a C-terminal HECT-type ubiquitin-protein ligase domain essential for the transfer of ubiquitin to the targeted substrate^19^,^20^,^21^. To date, Nedd4-2 is best known for its ability to regulate stability and activity of ion channels and transporters, particularly in epithelial cells^22^, but little is known about the role of this ubiquitin ligase in allergic inflammation. Recently, genetic studies from asthma-enriched families have identified a variant in *NEDD4L* associated with increased risk of the disease^23^. We have found that mast cells express Nedd4-2 and importantly, loss of Nedd4-2 in foetal liver-derived mast cells (FLMCs) or bone marrow-derived cultured mast cells (BMCMCs) not only results in heightened and sustained pro-inflammatory mediator release by mast cells *in vitro*, but also in prolonged IgE-mediated passive cutaneous anaphylaxis reactions in three different types of mast cell-deficient mice engrafted with *Nedd4-2*^−/−^ mast cells. Notably, we ascertained that the underlying mechanism involves phosphorylated (p)-Syk, but not p-Lyn, as a target of Nedd4-2-mediated ubiquitination and that the adapter molecule Ndfip1 (Nedd4 family interacting protein 1; refs 24, 25) participates in this process. These findings reveal that Nedd4-2 is an important intracellular gatekeeper in the control of mast cell-driven allergic inflammation and raise the possibility that alterations in this signalling pathway play a role in human disease.

## Results

### Nedd4-2 negatively regulates IgE-induced mediator release

**Figure 1:**
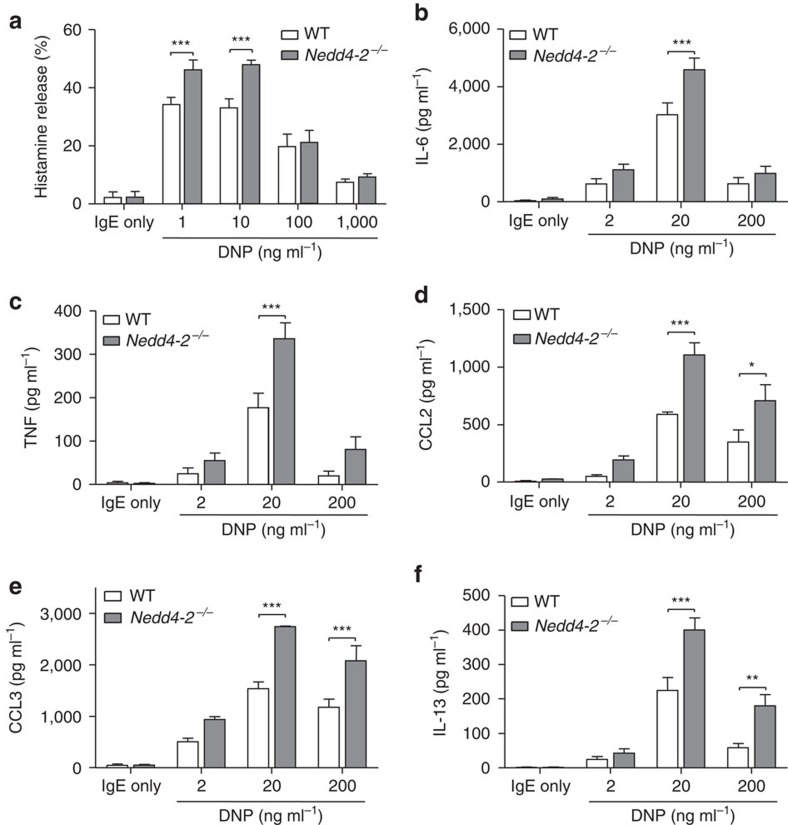
Loss of mast cell–Nedd4-2 enhances IgE-induced mediator release. WT and *Nedd4-2*^*−/−*^ FLMCs were sensitized with IgE anti-DNP antibody (clone SPE-7, 2 μg ml^−1^) for 16 h, then stimulated with indicated concentrations of DNP–HSA for measurement of release of (**a**) histamine (30 min), (**b**) IL-6, (**c**) TNF, (**d**) CCL2, (**e**) CCL3 and (**f**) IL-13 (**b**–**f** all 6 h). Data (mean±s.e.m.) are pooled from the three (**a**,**d**,**e**) or six (**b**,**c**,**f**) independent experiments performed, each of which gave similar results. *P<0.05, **P<0.01, ***P<0.001 for indicated comparisons (two-way analysis of variance (ANOVA) with Bonferroni post test).

To investigate the physiological function of mast cell Nedd4-2 we first confirmed that normal wild-type (WT; that is, *Nedd4-2*^*+/+*^) B6-mouse mast cells, derived from cultured bone marrow (BMCMCs) or embryonic (E18.5) foetal liver cells^26^ (FLMCs), express this ubiquitin ligase and that this is not true of mast cells derived from C57BL/6-*Nedd4-2*^*−/−*^ mice which exhibit a complete loss of Nedd4-2 expression (both mRNA and protein)^27^ (Supplementary Fig. 1a). Given the paucity in the number of surviving *Nedd4-2*^*−/−*^ mice postnatally^27^, we primarily used FLMCs, rather than BMCMCs, for our studies. We found that loss of Nedd4-2 in IgE-sensitised FLMCs activated by specific Ag (2,4-dinitrophenol-human serum albumin (DNP-HSA)) conferred a marked increase in the release of the pro-inflammatory mediators, histamine (1 and 10 ng ml^−1^ DNP for 30 min; Fig. 1a), IL-6, TNF, CCL2 and CCL3, as well as higher levels of the classic T_H_2 cytokine IL-13 at 6 h compared with WT littermate FLMCs (all with 20 ng ml^−1^ DNP and also with 200 ng ml^−1^ DNP for CCL2, CCL3, IL-13 only; Fig. 1b–f). Notably, the elevated release of IL-6 and TNF in IgE+Ag activated *Nedd4-2*^*−/−*^ FLMCs was sustained over a 20 h period for both concentrations of DNP used (2 and 20 ng ml^−1^; Supplementary Fig. 2a,b). These findings were not limited to the clone of IgE-anti DNP used (SPE7 versus H1-DNP-ɛ26, Fig. 1a–c and Supplementary Fig. 3a–c, respectively, for histamine, IL-6 and TNF release), or the progenitor source of the mast cell population (that is, foetal liver versus bone marrow; Fig. 1b,c and Supplementary Fig. 4a,b, respectively, for IL-6 and TNF), or if the FLMCs were generated with stem cell factor and IL-3 (Supplementary Fig. 5a,b) compared with IL-3 alone (Fig. 1b,c).

To ascertain if the enhanced IgE-mediated cytokine and histamine release by *Nedd4-2*^*−/−*^ mast cells was due to altered mast cell development, we assessed developmental parameters in cells cultured for 6 weeks, and found no distinction between *Nedd4-2*^*−/−*^ mast cells and their WT counterparts in cell surface expression of c-Kit and FcɛRI, cytoplasmic granule morphology by May Grünwald-Giemsa stain, numbers of mast cells generated in cultures seeded with 5 × 10^6^ foetal liver cells, expression of mRNA of mouse mast cell proteases 1, 2, 4, 5 and 6, or the ability of the mast cells to respond to IL-3-mediated signalling as determined by levels of phospho (p)-STAT5 and pErk (Supplementary Fig. 1b–e). Together, these data indicate that *Nedd4-2*^*−/−*^ mast cells appear to develop normally *in vitro* and that this ubiquitin ligase is required to restrain the extent and duration of IgE-mediated histamine and pro-inflammatory cytokine release from mast cells.

### Prolonged passive cutaneous anaphylaxis in *Nedd4-2* ^*−/−*^ mice

Mast cell activation is thought to contribute significantly to the pathogenesis of allergic diseases, such as anaphylaxis. To test the consequences associated with loss of mast cell-Nedd4-2 activity in the skin, we used a mast cell-dependent model of IgE-induced passive cutaneous anaphylaxis (PCA)^28^,^29^,^30^. The specific contribution of mast cell–Nedd4-2 in this setting was assessed using genetic and cell transfer approaches in three types of mast cell-deficient mice; namely mast cell-deficient B6-carboxypeptidase (*Cp)a3-Cre; Mcl-1*^*fl/fl*^ (commonly known as ‘Hello *Kit*ty' mice), which also have a modest deficiency in basophils)^28^, as well as the c-*kit* dysregulated C57BL/6J-*Kit*^W-sh/W-sh^ mice^31^,^32^ or mutant WBB6F_1_-*Kit*^W/W-v^ mice^4^,^31^. Each of these mice are profoundly mast cell deficient and can be selectively engrafted with *in vitro*-derived mast cells from genetically compatible WT mice or gene modified (for example, *Nedd4-2*^*−/−*^) mice. This ‘mast cell knock-in' approach has proven useful in dissecting the mechanisms of anaphylaxis^29^,^30^,^33^ as it reveals the extent to which mast cells can contribute to PCA reactions, separating these responses from those potentially caused by other phenotypic abnormalities in the Hello *Kit*ty^28^ mice (that is, basophil deficiency) or c-*kit* mutant mice (for example, inherent neutrophil abnormalities)^4^,^31^,^32^.

**Figure 2:**
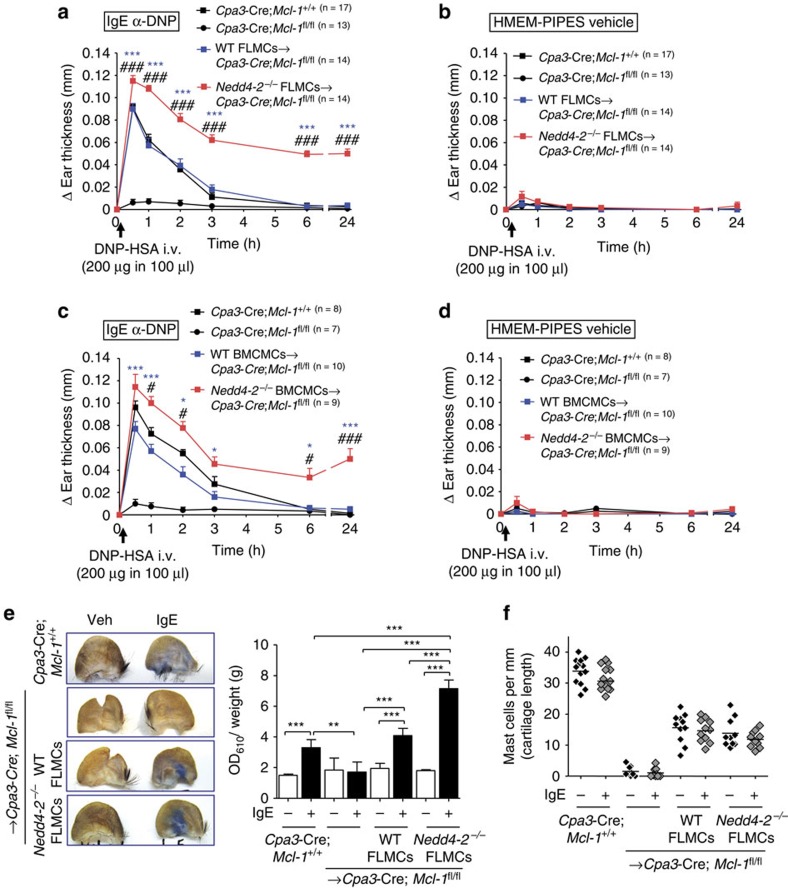
Mast cell–Nedd4-2 restrains IgE-mediated passive cutaneous anaphylaxis. Changes (Δ) in ear thickness 0–24 h after i.v. injection of DNP–HSA (200 μg in 100 μl) into mice, with DNP–HSA given 16 h after i.d. injection of anti-DNP IgE (SPE-7, 100 ng) in the right ear pinna (**a**,**c**) and equal volume of HMEM-Pipes vehicle in the left ear pinna (**b**,**d**) of *Cpa3-Cre*; *Mcl-1*^+/+^ (filled black squares), mast cell-deficient *Cpa3-Cre*; *Mcl-1*^fl/fl^ (filled black circles), and mast cell-deficient mice engrafted i.d. with (**a**,**b**) WT FLMCs (WT FLMCs→*Cpa3-Cre*; *Mcl-1*^fl/fl^, filled blue squares) or *Nedd4-2*^−/−^ FLMCs (*Nedd4-2*^−/−^ FLMCs→*Cpa3-Cre*; *Mcl-1*^fl/fl^, filled red squares), and (**c**,**d**) WT BMCMCs→*Cpa3-Cre*; *Mcl-1*^fl/fl^ (filled blue squares) or *Nedd4-2*^−/−^ BMCMCs→*Cpa3-Cre*; *Mcl-1*^fl/fl^ (filled red squares) mice. Data (mean±s.e.m.) are pooled from the four (**a**,**b**) or three (**c**,**d**) independent experiments performed, each of which gave similar results, each with 3–5 mice per group. *P<0.05, ***P<0.001 for comparisons of WT MCs versus *Nedd4-2*^−/−^ MCs→*Cpa3-Cre*; *Mcl-1*^fl/fl^ mice. ^#^*P*<0.05, ^###^*P*<0.001 for comparisons of WT mice versus *Nedd4-2*^−/−^ MCs→*Cpa3-Cre*; *Mcl-1*^fl/fl^ mice (two-way analysis of variance (ANOVA) with Bonferroni post test). (**e**) Evans blue dye extravasation (weight adjusted) quantified by absorption at 610 nm in vehicle or IgE anti-DNP treated ear pinnae at 30 min after i.v. (tail vein) DNP–HSA (containing Evans blue dye) administration. Representative ears shown for each group of mice tested. Data (mean±s.d.) are from one experiment with 4–5 mice per group. **P<0.01, ***P<0.001 for indicated comparisons (one-way ANOVA with Bonferroni post test); (**f**) Dermal mast cell numbers in ear pinnae of mice at the completion of three of the PCA experiments (that is, at 24 h after injection of DNP–HSA) outlined in (**a**,**b**).

**Figure 3:**
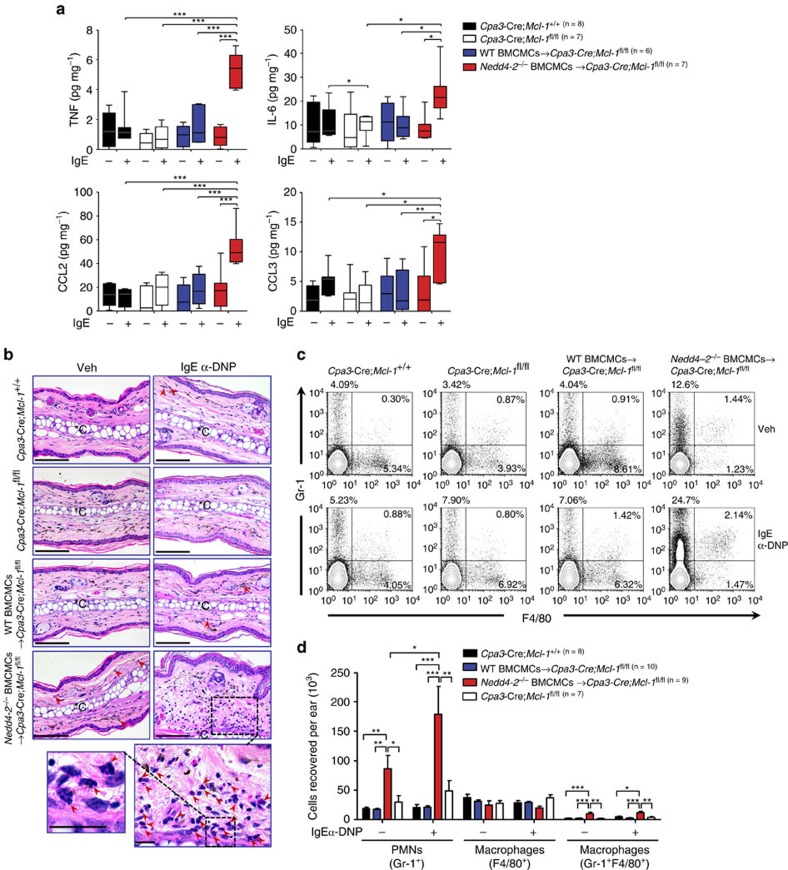
High inflammatory cytokines and sustained inflammation associated with passive cutaneous anaphylaxis in *Nedd4-2*^*−/−*^ mice. Ears injected i.d. with IgE anti-DNP or HMEM-pipes vehicle at 24 h after i.v. injection of DNP–HSA in *Cpa3-Cre*; *Mcl-1*^+/+^, mast cell-deficient *Cpa3-Cre*; *Mcl-1*^fl/fl^, WT BMCMCs→*Cpa3-Cre*; *Mcl-1*^fl/fl^, or *Nedd4-2*^−/−^ BMCMCs→*Cpa3-Cre*; *Mcl-1*^fl/fl^ mice. (**a**) Levels of TNF, IL-6, CCL2, and CCL3 in ear lysates; (**b**) H&E stained cross-sections of ears; (**c**) Representative flow cytometric plots; and (**d**) Cells recovered per ear of gated populations of polymorphonuclear (PMN) leukocytes (Gr-1^+^F4/80^−^) and macrophages (Gr-1^−^F4/80^+^ and Gr-1^+^F4/80^+^) (**b**) *C, cartilage. Red arrowheads indicate PMNs. Scale bars: 100 μm (insets 20 μm). Percentage values in **c** refer to percentage of total viable cells present in the depicted section of the plot. Data (**a**, median with interquartile ranges) and (**d**, mean±s.e.m.) are pooled from two (**a**) or three (**d**) independent experiments performed, each of which gave similar results, each with 3–5 mice per group. *P<0.05, **P<0.01, ***P<0.001 for indicated comparisons (one-way analysis of variance (ANOVA) with Bonferroni (**a**) or Dunnett's (**d**) post test).

As previously described^28^, mast cell-sufficient *Cpa3-Cre; Mcl-1*^*+/+*^ mice developed tissue swelling that peaked at 30 min and resolved by 6 h after Ag challenge, whereas the mast cell-deficient *Cpa3-Cre; Mcl-1*^*fl/fl*^ mice exhibited weak reactions (Fig. 2a,c), with swelling similar to the vehicle-injected ear pinnae of all the groups of mice tested (Fig. 2b,d). By contrast, *Cpa3-Cre; Mcl-1*^*fl/fl*^ mice engrafted intra-dermally (i.d.) in their ear pinnae with *Nedd4-2*^*−/−*^ FLMCs (Fig. 2a) or *Nedd4-2*^*−/−*^ BMCMCs (Fig. 2c) exhibited enhanced IgE-Ag-mediated vascular permeability as evidenced by increased Evans blue dye extravasation in the IgE-sensitised ear skin (Fig. 2e; FLMCs only), together with strikingly prolonged anaphylactic responses lasting >24 h after IgE-Ag challenge), compared with reactions which resolved within 6 h in the WT mast cell-engrafted groups and the corresponding control *Cpa3-Cre; Mcl-1*^*+/+*^ mice (Fig. 2a,c). At the 24 h time point, we also observed in the IgE-sensitized ears of the *Nedd4-2*^*−/−*^ BMCMC-engrafted mice elevated levels of the pro-inflammatory mediators TNF, IL-6, CCL2 and CCL3 (Fig. 3a) that correlated with a distinct Gr-1^+^ polymorphonuclear (PMN) cell infiltration, as determined by H&E staining of the tissues and flow cytometric analyses (Fig. 3b–d). Interestingly, elevated numbers of PMNs (but not to the same extent as in the PCA-induced PMN cell infiltration in the IgE-injected ears), were also observed in the vehicle-treated ears of the *Nedd4-2*^*−/−*^ BMCMC-engrafted mice. By contrast, there were no differences in the thickness of the ear pinnae at baseline between vehicle-treated or IgE-treated ears before Ag-DNP injection in any of the mouse groups tested, and no change in ear thickness above baseline at the 24 h time point was detected in the IgE- and Ag-DNP-induced PCA reactions in any of the groups examined (Fig. 3c,d and Fig. 2d). This suggests that whatever effects *Nedd4-2*^*−/−*^ mast cells may have had in such vehicle-injected ears which influenced numbers of PMNs at these sites, these were not sufficient to induce substantial increases in local vascular permeability, and therefore were unlikely to have been associated with substantial mast cell degranulation. However, given the notable negative regulatory role of Nedd4-2 on mast cell activation demonstrated in our study, it is possible that mast cells lacking Nedd4-2 can exhibit an inherent propensity for over-activity that can occur independently of their direct activation via IgE+specific Ag (DNP–HSA) during PCA responses, such as upon local injection of vehicle into the ear pinna. Indeed, we cannot exclude the possibility that, in the *in vivo* setting, mast cell-Nedd4-2 deficiency can influence other aspects of mast cell phenotype and function beyond those directly examined in this study.

The biological significance of the PCA findings observed in the mast cell-deficient *Cpa3-Cre; Mcl-1*^*fl/fl*^ mice was corroborated in experiments performed with two other types of mast cell-deficient mice, C57BL/6J-*Kit*^W-sh/W-sh^ and WBB6F_1_-*Kit*^W/W-v^ mice where, notwithstanding the particular range of abnormalities carried by each, a similarly pronounced PCA reaction was observed in each of the *Nedd4-2*^*−/−*^ FLMCs groups (Supplementary Fig. 6a,c). Furthermore, the differences between the IgE-Ag-challenged WT or *Nedd4-2*^*−/−*^ FLMC groups were unlikely to be related to disparities in the extent of mast cell engraftment because similar numbers of ear pinna mast cells were present in the two groups, irrespective of IgE sensitization (Fig. 2f; Supplementary Fig. 6e). These data support the conclusion that a loss of mast cell-Nedd4-2 during IgE-mediated PCA leads to sustained mast cell-dependent inflammation (likely reflecting sustained mast cell activation in this setting) with significant biological consequences *in vivo*.

### *Nedd4-2* ^*−/−*^ mast cells show enhanced FcɛRI-mediated signalling

**Figure 4:**
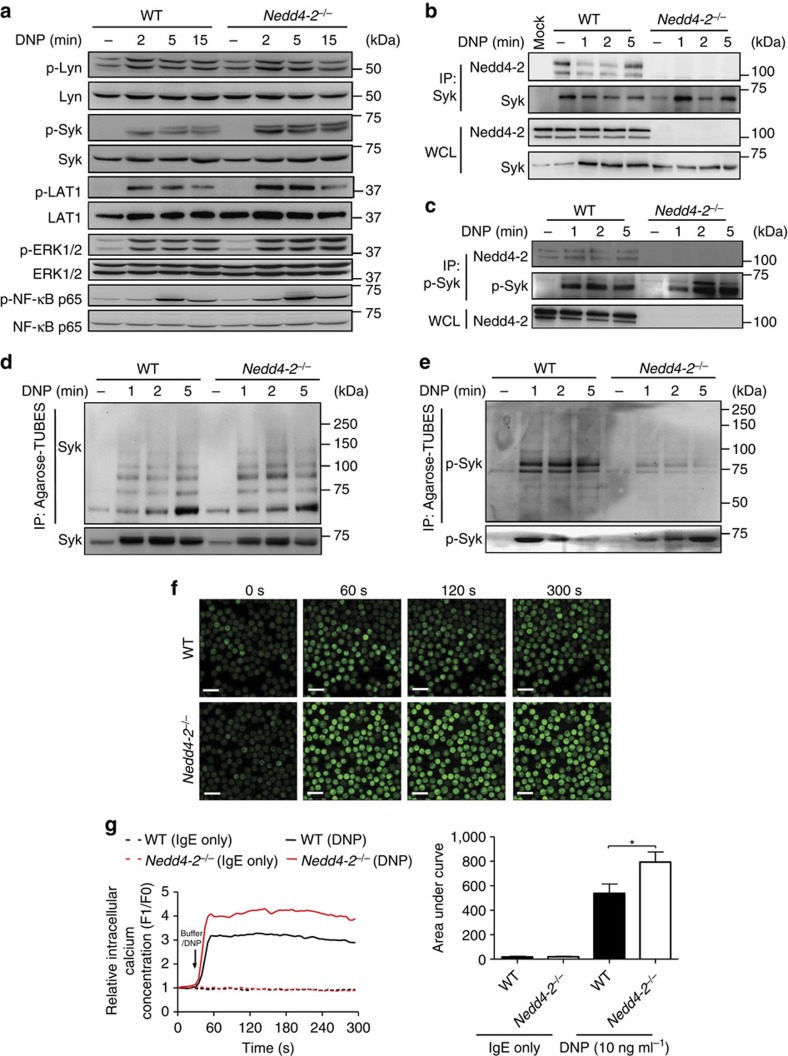
Nedd4-2 interacts with p-Syk and mediates its ubiquitination in mast cells. (**a**) Immunoblot analysis of phosphorylated (p-) and total signalling Lyn, Syk, LAT1, ERK1/2 and NF-κB-p65 proteins in whole cell lysates prepared from IgE anti-DNP (SPE-7; 2 μg ml^−1^) sensitized WT or *Nedd-4-2*^−/−^ FLMCs stimulated with DNP–HSA (20 ng ml^−1^) for the indicated time points. Immunoblot analysis of Nedd4-2 immunoprecipitated (IP) with Syk (**b**) and p-Syk (**c**) and whole-cell lysates of FLMCs prepared and stimulated with DNP–HSA as in **a**. IP: MOCK indicates an IP control performed without antibody. Ubiquitylation of Syk (**d**) and p-Syk (**e**) using Agarose-Tandem Ubiquitin Binding Entities in cell extracts from WT and *Nedd4-2*^−/−^ FLMCs. Data are representative of the three (**a**–**e**) independent experiments performed, each of which gave similar results. (**f**) Representative confocal micrographs of intracellular calcium influx over indicated times in IgE anti-DNP (SPE-7, 2 μg ml^−1^) sensitized WT and *Nedd4-2*^−/−^ FLMCs incubated with Fluo-3 (5 μM) for 30 min before addition of DNP–HSA (10 ng ml^−1^). Scale bars, 20 μm. (**g**) Representative experiment of DNP induced-Ca^2+^ influx in WT versus *Nedd4-2*^−/−^ FLMCs (left panel) and quantified analyses of area under the Ca^2+^ influx curves (right panel). Arrow indicates time of DNP–HSA addition to the cells. Data (right panel, mean±s.e.m.) are pooled from the four independent experiments performed, each of which gave similar results. *P<0.05 for indicated comparison (one-way analysis of variance (ANOVA) with Bonferroni post test).

The data presented above strongly suggest that impaired Nedd4-2 function in mast cells leads to alterations in intracellular signalling events downstream of IgE-FcɛRI aggregation. To define the underlying mechanisms, we first evaluated the impact of Nedd4-2 deficiency on FcɛRI-mediated *Src* related kinase and MAPK activation kinetics, and examined the phosphorylation of FcɛRI-proximal molecules, Lyn, Syk and LAT1. Although pLyn levels were unchanged over the time courses studied, phosphorylation of Syk and to a lesser extent LAT1 were increased (Fig. 4a; Supplementary Fig. 7a,b), in *Nedd4-2*^*−/−*^ FLMCs versus WT FLMCs. MAPK cascades as well as certain transcription factors, such as NF-κB, orchestrate the production of IgE-induced cytokines from mast cells^8^,^16^,^34^ and are downstream of Lyn, Syk and LAT1/2 activation. We found that FcɛRI-mediated phosphorylation of the MAPKs JNK1/2 and p38 were unaltered by the absence of Nedd4-2, whereas p-ERK1/2 was significantly elevated at 5 and 10 min after stimulation in *Nedd-2*^−/−^ FLMCs compared to WT FLMCs (Fig. 4a; Supplementary Fig. 7a,b). In the same experimental setting, phosphorylated NF-κB-p65 was also enhanced between 2 and 15 min post Ag stimulation (Fig. 4a; Supplementary Fig. 7a,b). We also assessed calcium mobilization, a key regulator of degranulation^35^, in IgE+cognate Ag-stimulated FLMCs. *Nedd4-2*^*−/−*^ FLMCs showed a sharp cytosolic Ca^2+^ spike followed by a sustained Ca^2+^ plateau, whereas cytosolic Ca^2+^ influx was considerably lower in WT FLMCs (Fig. 4f,g); findings consistent with the enhanced histamine release of *Nedd4-2*^*−/−*^ FLMCs relative to WT FLMCs (Fig. 1a; Supplementary Fig. 3a). Collectively, these findings provided us with an important clue as to the function of Nedd4-2 in this setting, suggesting that the *Src* tyrosine kinase, Syk, upstream of the ERK1/2 and NF-κB pathways and Ca^2+^ mobilization, might be a target of Nedd4-2 ubiquitination.

### p-Syk is a substrate of Nedd4-2-mediated ubiquitination

Because Nedd4-2 deficiency leads to heightened p-Syk and in turn propagation of stronger signals downstream of this activating kinase following Ag-induced IgE-FcɛRI aggregation, we first evaluated the association of Syk and Nedd4-2 in mast cells. In line with Syk being a *bona fide* substrate of Nedd4-2, both total Syk (Fig. 4b) and p-Syk (Fig. 4c; Supplementary Fig. 3d) immunoprecipitated with Nedd4-2 in mast cells generated from WT mice but not *Nedd4-2*^−/−^ mice. Interestingly, in IgE-sensitized WT FLMCs without Ag stimulation, Nedd4-2 appeared to associate with p-Syk, an interaction that might occur to negatively regulate the cytokinergic activity of the SPE-7 IgE used in these experiments when bound to FcɛRI^36^,^37^. To address the possibility that Nedd4-2 targets Syk for degradation, FLMCs were stimulated with IgE+DNP–HSA, and we searched by co-immunoprecipitation and immunoblotting for evidence of a physical interaction between Syk (both total and phosphorylated forms) and ubiquitin. Although p-Lyn and total Syk appeared to be equally ubiquitinated (Supplementary Fig. 8; Fig. 4d) in both WT and *Nedd4-2*^−/−^ FLMCs, we observed substantially reduced polyubiquitination of p-Syk in *Nedd4-2*^−/−^ FLMCs, particularly at 1–5 min after Ag stimulation in comparison to the WT counterparts (Fig. 4e). Thus, we concluded that the elevated levels of p-Syk (Fig. 4a) resulting in subsequent elevated and prolonged mediator release (Fig. 1a–f; Supplementary Figs 2a,b, 3a–c, 4a,b and 5a,b) in IgE-activated *Nedd4-2*^−/−^ FLMCs or BMCMCs is due to the lack of Nedd4-2 binding and ubiquitination of p-Syk.

### Ndfip1 restrains FcɛRI-mediated signalling

**Figure 5:**
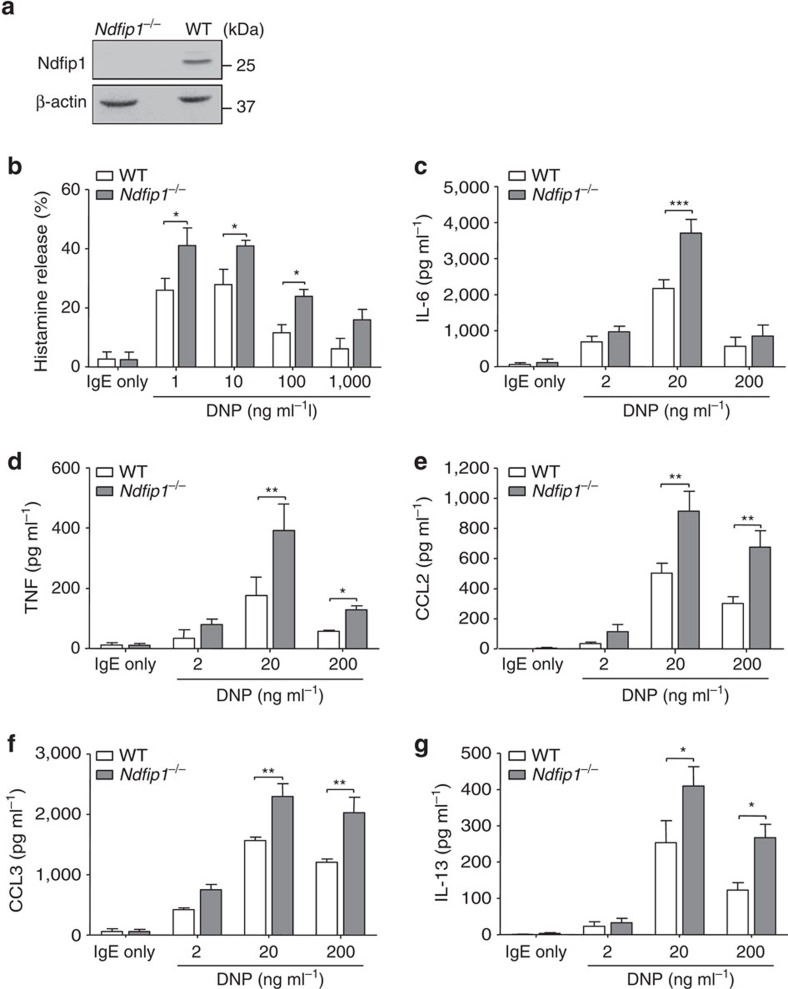
Loss of mast cell–*Ndfip1*^*−/−*^ enhances IgE-induced mediator release. (**a**) Immunoblot analysis of Ndfip1 expression in WT or *Ndfip1*^*−/−*^ BMCMCs. (**b**–**g**) WT and *Ndfip1*^*−/−*^ BMCMCs sensitized with IgE anti-DNP antibody (SPE-7, 2 μg ml^−1^) for 16 h, then stimulated with indicated concentrations of DNP–HSA for measurements of the release of (**b**) histamine (30 min), (**c**) IL-6, (**d**) TNF, (**e**) CCL2, (**f**) CCL3 and (**g**) IL-13 (**c**–**g** all 6 h). Data (mean±s.e.m.) are pooled from the three independent experiments performed, each of which gave similar results. *P<0.05, **P<0.01, ***P<0.001 for indicated comparisons (two-way analysis of variance (ANOVA) with Bonferroni post test).

**Figure 6:**
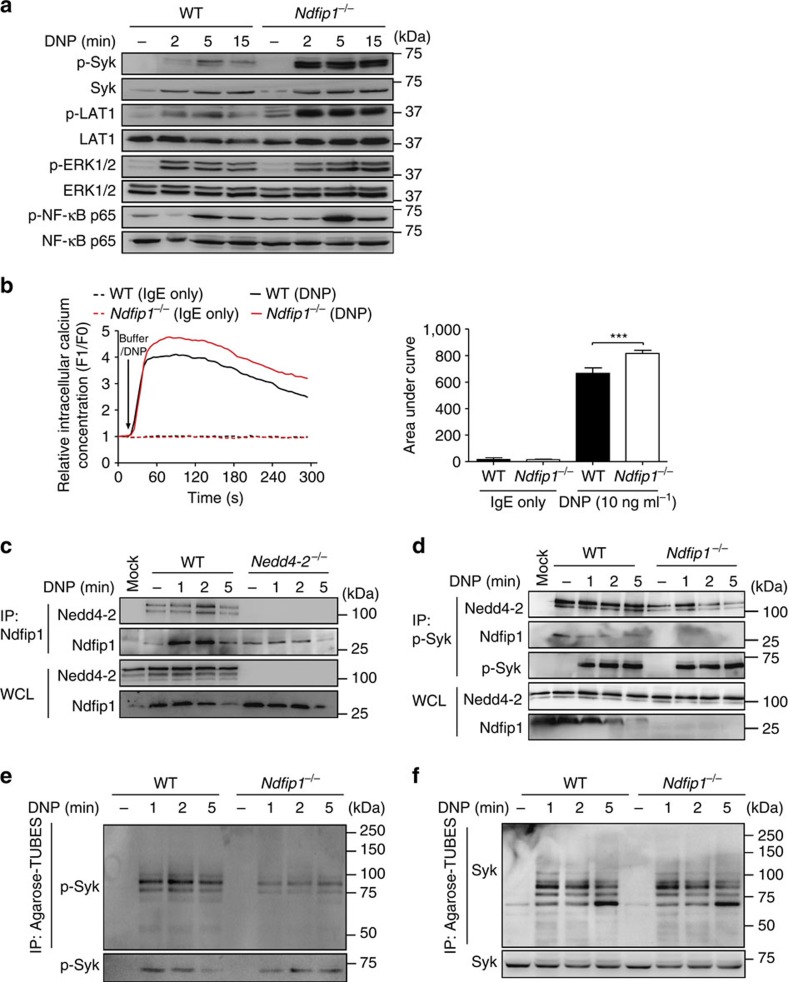
Ndfip1 is an adaptor protein for Nedd4-2 function in mast cells. (**a**) Immunoblot analysis of phosphorylated (p-) and total signalling Lyn, Syk, LAT1, ERK1/2 and NF-κB-p65 proteins in whole cell lysates prepared from IgE anti-DNP (SPE-7, 2 μg ml^−1^) sensitized WT or *Ndfip1*^−/−^ BMCMCs stimulated with DNP–HSA (20 ng ml^−1^) for the indicated time points. (**b**) Representative experiment of DNP induced-Ca^2+^ influx in WT versus *Ndfip1*^−/−^ FLMCs (left panel) and quantified analyses of area under the Ca^2+^ influx curves (right panel). Arrow indicates time of DNP–HSA addition to the cells. Data (mean±s.e.m.) are pooled from the four independent experiments performed, each of which gave similar results. ***P<0.001 for indicated comparison (one-way analysis of variance (ANOVA) with Bonferroni post test). Immunoblot analysis of (**c**) Nedd4-2 immunoprecipitated (IP) with anti-Ndfip1 from WT and *Nedd4-2*^−/−^ FLMCs, and (**d**) Nedd4-2 and Ndfip1 immunoprecipitated with anti-p-Syk from WT and *Ndfip1*^−/−^ BMCMCs prepared and stimulated with DNP–HSA as in **a**. above for indicated times. IP: MOCK indicates an IP control performed without antibody. Ubiquitylation of p-Syk (**e**) and Syk (**f**) using Agarose-Tandem Ubiquitin Binding Entities in cell extracts from WT and *Ndfip1*^−/−^ BMCMCs. Data are representative of the three (**c**–**f**) independent experiments performed, each of which gave similar results.

The PPxY (PY) or similar proline-rich motifs in the substrate are required for direct binding to the WW domains of Nedd4-2. Since the Syk protein sequence lacks such binding motifs, we investigated whether an adaptor protein such as Ndfip1 (Nedd4 family interacting protein 1) was responsible for binding to p-Syk and augmenting the function of Nedd4-2 (refs 20, 21, 38). Mast cells express Ndfip1 (Fig. 5a) and in its absence, *Ndfip1*^−/−^ BMCMCs elicited responses similar to those induced in IgE+Ag-activated Nedd4-2-deficient mast cells (Fig. 1a–f; Supplementary Figs 4a,b and 5a,b), including elevated histamine release at the 30 min time point (Fig. 5b), as well as marked increases in the production of IL-6, TNF, CCL2, CCL3 and IL-13 at 6 h, particularly for the highest concentrations of Ag used (Fig. 5c–g). In accordance with these results, IgE-activated *Ndfip1*^−/−^ mast cells displayed amplified phosphorylation of Syk, LAT1, Erk1/2 and NF-κB p65 (Fig. 6a), as well as heightened cytosolic Ca^2+^ influx (Fig. 6b) compared with the WT counterparts. Immunoprecipitation confirmed that Ndfip1 physically interacts with Nedd4-2 (Fig. 6c) and p-Syk (Fig. 6d) in WT BMCMCs but not in *Ndfip1*^−/−^ BMCMCs. In contrast to WT BMCMCs, *Ndfip1*^−/−^ BMCMCs-like *Nedd4-2*^−/−^ FLMCs (Fig. 4e), exhibited markedly lower polyubiquitination of p-Syk (Fig. 6e) but no difference in total Syk ubiquitination (Fig. 6f) after Ag stimulation at 1 to 5 min. However, it should be noted that some interaction between Nedd4-2 and p-Syk, albeit significantly reduced compared with WT BMCMCs, was detected in the *Ndfip1*^−/−^ BMCMCs (Fig. 6d), indicating that although Ndfip1 is required to mediate optimal functional interaction of Nedd4-2 with p-Syk, other binding partners that bind to both Nedd4-2 and p-Syk are also present in the complex in the absence of Ndfip1.

**Figure 7:**
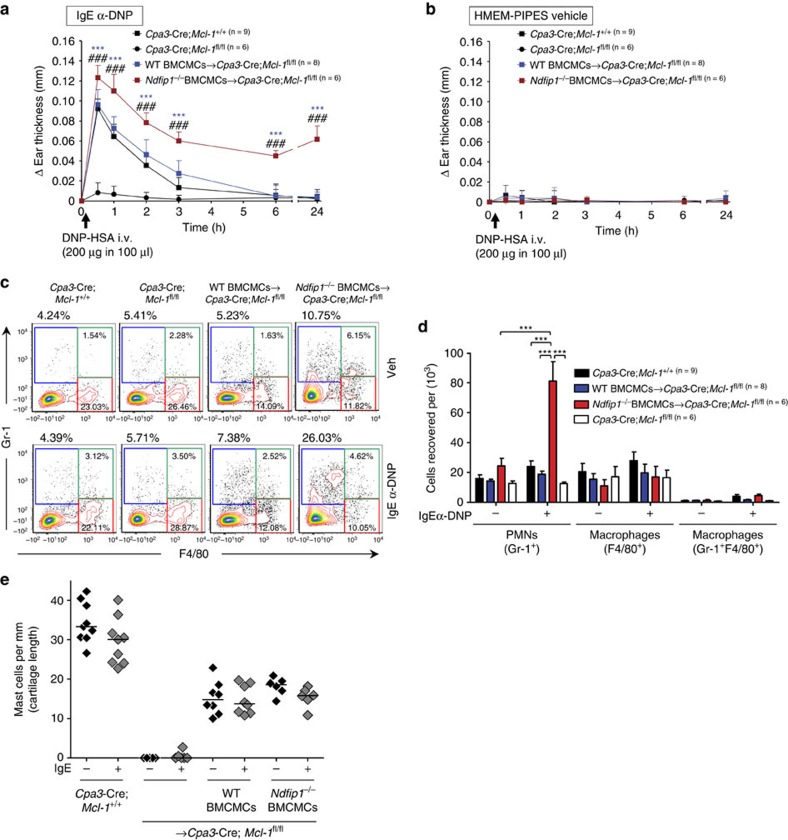
Mast cell–Ndfip1 restrains IgE-mediated passive cutaneous anaphylaxis. Changes (Δ) in ear thickness 0–24 h after i.v. injection of DNP–HSA (200 μg in 100 μl) into mice, with DNP–HSA given 16 h after i.d. injection of (**a**) anti-DNP IgE (SPE-7, 100 ng) in the right ear pinna and (**b**) equal volume of HMEM-Pipes vehicle in the left ear pinna of (**a**,**b**) *Cpa3-Cre*; *Mcl-1*^+/+^ (filled black squares), mast cell-deficient *Cpa3-Cre*; *Mcl-1*^fl/fl^ (filled black circles), and mast cell-deficient mice engrafted i.d. with WT BMCMCs (WT BMCMCs→*Cpa3-Cre*; *Mcl-1*^fl/fl^, filled blue squares) or *Ndfip1*^−/−^ BMCMCs (*Ndfip1*^−/−^ BMCMCs→*Cpa3-Cre*; *Mcl-1*^fl/fl^, filled red squares). (**c**) Representative flow cytometric plots; and (**d**) Cells recovered per ear of gated populations of polymorphonuclear (PMN) leukocytes (Gr-1^+^F4/80^−^) and macrophages (Gr-1^−^F4/80^+^ and Gr-1^+^F4/80^+^). Percentage values in **c** refer to percentage of CD45^+^ viable cells present in the depicted section of the plot. (**e**) Dermal mast cell numbers in ear pinnae of mice at the completion the PCA experiments (that is, at 24 h after injection of DNP–HSA) outlined in (**a**,**b**). (**a**,**b**,**d**) Data (mean±s.d.) are pooled from the two independent experiments performed, each of which gave similar results, each with 3–5 mice per group. (**a**,**b**) ***P<0.001 for comparisons of WT BMCMCs versus *Ndfip1*^−/−^ BMCMCs→*Cpa3-Cre*; *Mcl-1*^fl/fl^ mice. ^###^*P*<0.001 for comparisons of WT mice versus *Ndfip1*^−/−^ MCs→*Cpa3-Cre*; *Mcl-1*^fl/fl^ mice (two-way analysis of variance (ANOVA) with Bonferroni post test). (**d**) ***P<0.001 for indicated comparisons (one-way ANOVA with Bonferroni post test).

Finally, we set out to determine if loss of mast cell–Ndfip1 caused similar IgE-mediated PCA reactions and pathology as those we observed when Nedd4-2 was absent in skin mast cells (Fig. 2a,c). A sustained anaphylactic response with a Gr-1^+^ PMN cell infiltrate into the IgE-treated ears characterised the 24 h time point in the *Cpa3-Cre; Mcl-1*^*fl/fl*^ mice engrafted with *Ndfip1*^−/−^ BMCMCs (Fig. 7a–d). Furthermore, similar to the findings in *Nedd4-2*^−/−^ mast cell engrafted mast cell-deficient mice (Fig. 2a,c; Supplementary Fig. 6a,c), differences in the PCA reactions were not due to disparities in mast cell numbers in the WT or *Ndfip1*^−/−^ BMCMC engrafted groups (Fig. 7e).

Taken together, these data indicate that this E3 ligase adaptor is required for optimal bridging of p-Syk with Nedd4-2; a function that appears to facilitate maximal negative regulation of p-Syk in the IgE/FcɛRI signal transduction pathway and subsequently controls mast cell mediator release to limit excessive reactions and pathology *in vivo*.

## Discussion

Our findings have identified previously unknown functions for the ubiquitin ligase Nedd4-2 and its adaptor Ndfip1 in the negative intracellular regulation of the IgE/FcɛRI signal transduction pathway in mast cells. We have shown that loss of p-Syk negative regulation by Nedd4-2/Ndfip1 proximal to FcɛRI aggregation results in heightened and prolonged propagation of multiple downstream pathways including the MAP kinase cascade, involving the ERK1/2, NF-κB pathway and calcium mobilization. This latter process is essential for mast cell degranulation and histamine release. Collectively, our data demonstrate that the Nedd4-2/Ndfip1 axis in mast cells represents a previously unknown mechanism contributing to the control of the magnitude and duration of inflammatory mediator release. The loss of mast cell-Nedd4-2/Ndfip1 activity is significant biologically, as it results in exacerbated mediator release *in vitro* and sustained IgE-mediated anaphylaxis-associated inflammation *in vivo*.

It is well recognized that Syk is essential for amplification of the signals required for mast cell function as loss of its tyrosine kinase activity results in diminished calcium responses, degranulation and cytokine production following FcɛRI stimulation^39^. By contrast, prolonged phosphorylation of Syk and its substrates augments histamine release^40^ and markedly elevates production of the proinflammatory cytokines TNF, IL-6 and CCL2 (ref. 41). Thus, tight control of the FcɛRI signalosome is essential to guard against the pathological consequences associated with sustained mast cell activation. Syk activation is thought to be governed in part by dephosphorylation of phospho-Tyr^58^ in the ITAM domain of FcRγ via receptor-associated phosphatases SHP-1 and SHP-2 (ref. 42), as well as by Cbl-mediated ubiquitylation and degradation^40^,^41^.

Studies in normal and ‘non-releaser' human basophils have indicated that loss of Syk expression that leads to negative regulation (1–18 h after stimulation)^43^ of, or absent^44^, IgE-induced basophil activation is caused by a proteasome-dependent mechanism involving c-Cbl-mediated Syk ubiquitination^43^. Interestingly, in B cells, eosinophils and neutrophils from ‘non-releaser' donors, Syk and Lyn appear to be expressed normally, indicating that in other leukocyte populations Syk expression is regulated differently compared with basophils^44^. With respect to mast cells it has been reported that *CBLB*^−/−^ BMCMCs, but not *CBLC*^−/−^ BMCMCs^41^, exhibit enhanced FcɛRI and Syk phosphorylation. Based on these findings it is of interest that Cbl-b RING finger mutant mast cells do not display retarded FcɛRI internalization nor the very high cytokine levels evident in *CBLB*^−/−^ BMCMCs^45^. This suggests that Cbl-b's negative regulation of FcɛRI signalling is largely independent of its E3 ligase activity; a finding supported by the work of Zhang *et al*.^40^, who failed to detect any increased Syk ubiquitination in WT BMCMCs compared with *CBLB*^−/−^ cells. It is possible that Cbl-b functions as an adaptor and/or docking molecule in this setting to facilitate the physical interaction between FcɛRI/Syk and other yet to be identified E3 ligases or phosphatases. Although it is unknown whether Nedd4-2/Ndfip1 interact with the Cbl family of proteins in mast cells, our data demonstrate that Nedd4-2 ubiquitinates phosphorylated Syk within minutes of stimulation, without altering total Syk expression over the same time frame, and for this to occur optimally Ndfip1 is required. Notably, the absence of either Nedd4-2 or Ndfip1 in mast cells results in elevated and sustained IgE-induced pro-inflammatory mediator release.

Our results are consistent with experimental studies investigating the role of Ndfip1 in autoimmune and allergic diseases. Mice that lack Ndfip1 naturally develop severe T_H_2-mediated inflammation in the skin, gut and lungs, exhibit high levels of circulating IgE and die prematurely^46^,^47^. To date, the contribution of mast cells to the pathology in these mice is unknown but studies focusing on T cells have identified that activated T_H_2-polarized CD4^+^ T cells are a feature, possibly due to elevated production of IL-2, IL-4 and IL-5 (refs 46, 48), as well as an inability to exit the cell cycle to abort T-cell clonal expansion in response to self and exogenous antigens^49^. Although loss of Ndfip1 is thought to impede Itch-mediated ubiquitination and degradation of the transcription factor JunB in this setting^46^, it is plausible that Nedd4-2 might also be required. Selective deletion of SGK1 in T cells has been shown to protect against pathology associated with an experimental model of allergic asthma by enhancing JunB ubiquitination in CD4^+^ T cells via a mechanism thought to involve Nedd4-2 and its adaptor Ndfip1 (ref. 50). Thus, in *Ndfip1*^−/−^ mice both Itch and Nedd4-2 activity would be disrupted in cell lineages where each of these ligases are selectively expressed and Ndfip1 is required to bind to, and to present such proteins for ubiquitination. It is unknown if Itch is expressed in mast cells, nevertheless our findings are the first to reveal that both Ndfip1 and Nedd4-2 are necessary to physiologically restrain FcɛRI signal transduction upon IgE-mediated mast cell activation *in vitro* and in the experimental setting of passive cutaneous anaphylaxis *in vivo*. These findings provide the context for future investigations in models of chronic allergic inflammation of the airways in which mast cells contribute to multiple features of the pathology^51^. Disruption of the Nedd4-2/Ndfip1 axis in mast cells would likely unleash multiple cycles of sustained antigen-IgE:FcɛRI aggregation, potentially driving an earlier onset of exacerbated pathology.

In summary, we have identified previously unknown functions of Nedd4-2 and Ndfip1 in the repertoire of inhibitory molecules expressed by mast cells that govern restraint of IgE-mediated activation. Our findings are likely to be relevant to the pathogenesis of human diseases in which mast cells are implicated. Human genetic studies have associated variants in *NDFIP1* with asthma^52^ and other inflammatory diseases (that is, rheumatoid arthritis^53^ and inflammatory bowel disease^54^). More recently, in a whole-genome sequencing study on individuals from a Hutterite population, a 6 kbp deletion in an intron in *NEDD4L* has been associated with increased risk of asthma^23^. Our experimental findings here, raise the possibility that such genetic alterations perturb the ability of Nedd4-2 to negatively regulate mast cell signalling, as well as that of other cell populations expressing this ligase and adaptor^50^, resulting in sustained inflammatory responses and a contribution to the sequelae of allergic disease in affected individuals. Our data also support the notion that novel therapeutic interventions to control allergic inflammation would benefit from targeting the Nedd4-2/Ndfip1 pathway to enhance Nedd4-2 activity.

## Methods

### Mice

Male and female *Nedd4l*-targeted B6 Nedd4-2-deficient (*Nedd4-2*^−/−^) and *Ndfip1*-targeted B6 Ndfip1-deficient (*Ndfip1*^−/−^) mice were generated as described in Boase *et al*.^27^ and Oliver *et al*.^46^, respectively. *Cpa3-Cre; Mcl-1*^*fl/fl*^ mice are severely deficient in mast cells and also have a marked deficiency in basophils^28^. In these mice, Cre recombinase is expressed under the control of the carboxypeptidase A3 (*Cpa3*) promoter. Mcl-1 is an intracellular anti-apoptotic protein that is required for mast cell survival. C57BL/6- *Cpa3-Cre; Mcl-1*^*+/+*^ mice were used as WT controls for *Cpa3-Cre; Mcl-1*^*fl/fl*^ mice. Genetically c-*kit* mutant mast cell-deficient (WB/ReJ-*Kit*^*W/+*^ × C57BL/6-*Kit*^*W-v/+*^)F_1_-*Kit*^*W/W-v*^ (WBB6F_1_-*Kit*^*W/W-v*^) (*Kit*^*W/W-v*^) mice and the congenic normal WBB6F_1_-*Kit*^*+/+*^ (*Kit*^*+/+*^) mice, were obtained from The Jackson Laboratory (Bar Harbor, Maine, USA) and bred in house. B6-*Kit*^W-sh/+^ mice, backcrossed with C57BL/6J (B6J) mice for 12 generations were used as breeding pairs to produce genetically mast cell-deficient B6J-*Kit*^W-sh/Wsh^ mice^55^. As previously reported, adult *Kit*^W-sh/Wsh^ and *Kit*^W/W-v^ mice have a profound deficiency of mast cells, with <1.0% the WT level of mast cells in the dermis^1^,^4^,^31^. For all *in vivo* experiments, age-matched male mice of 6–12 weeks of age were used and all mice were bred in house at the SA Pathology Animal Resource Facility (Adelaide, Australia). Experiments were performed in compliance with the ethical guidelines of the National Health and Medical Research Council of Australia, with approval from the SA Pathology/CALHN Animal Ethics Committee (South Australia).

### Generation of mouse FLMCs and BMCMCs

WT and *Nedd4-2*^−/−^ FLMCs and BMCMCs were obtained by culturing progenitor stem cells from foetal livers of E18.5 mice, and bone marrow cells from the femurs and tibias of 18–21-day old B6-WT or B6-*Nedd4-2*^*−/−*^ mice, in DMEM (Gibco) supplemented with 10% fetal calf serum (FCS; Bovogen) and 20% WEHI-3 conditioned medium (supplemented to 3–4 ng ml^−1^ IL-3 with recombinant mouse IL-3 (Shenandoah Biotechnology)) for 5–7 weeks. B6-WT and B6-*Ndfip1*^*−/−*^ BMCMCs were derived from the femoral bone marrow cells and cultured according to the aforementioned conditions. After 5 weeks of culture, >95% of the cells were identified as mast cells by May Grünwald-Giemsa staining and by flow cytometric analysis (c-Kit^+^ FcɛRI^+^)^1^,^2^,^29^.

### Antibody production and affinity purification

Anti-DNP mouse IgE monoclonal antibodies (clones SPE-7 and H1-DNP-ɛ-26) were affinity-purified by DNP/BSA column chromatography. Briefly, IgE-mAb-producing hybridoma cells (SPE-7 clone provided by Z. Eshar, Weizmann Institute of Science, Israel; H1-DNP-ɛ26 clone provided by F.-T. Liu, University of California-Davis, USA) were cultured in DMEM supplemented with 10% FCS at 10^5^ cells per ml for 4 days. Culture supernatant was purified with DNP-BSA packed HiTrap NHS-activated HP column (Amersham Biosciences) using an AKTApurifer system (GE Healthcare Life Sciences). Fractions collected were first concentrated using a Vivaspin 20 tube (Sartorius), and then dialysed in PBS. Purified SPE-7 and H1-DNP-ɛ-26 mAbs were quantified using the SMART system with a Superdex 200PC 3.2/30 column (Amersham Biosciences).

### Histamine and cytokine measurement

Five to seven week-old FLMCs or BMCMCs cultured in DMEM containing 10% FCS and 20% WEHI-3 conditioned medium (supplemented to 3 ng ml^−1^ IL-3 as outlined above) were sensitized for 16 h at 37 °C with IgE anti-DNP mAb (2 μg ml^−1^; generated from supernatants induced by the hybridomas SPE-7 clone or H1-DNP-ɛ-26 clone (where indicated), which produce different clones of an IgE mAb to DNP). For measurement of histamine release, after IgE sensitization FLMCs (10^6^ cells per ml) were re-suspended in Tyrode's buffer (129 mM NaCl, 8.4 mM glucose, 10 mM HEPES, 5 mM KCl, 1 mM MgCl_2_, 1.4 mM CaCl_2_ and 1% BSA at pH 7.4), aliquoted into polystyrene test tubes and then activated with 1–1,000 ng ml^−1^ DNP–HSA-specific antigen (30–40 DNP conjugated to each molecule of HSA (DNP_30-40_–HSA); Sigma-Aldrich) for 30 min at 37 °C. The reaction was stopped by the addition of ice-cold buffer followed immediately by centrifugation at 180*g* for 5 min at 4 °C. Cell pellets and supernatants were separated by transferring the supernatant in each tube into a new tube. Cell pellets were lysed with 0.5% Triton X-100 (Sigma-Aldrich) in Tyrode's buffer. Histamine levels in supernatants and cell lysates were measured using an EIA histamine kit (Beckman Coulter) according to the manufacturer's instructions. Histamine release was expressed as a percentage of total cellular content of histamine (histamine release (%)). For measurement of cytokine production, IgE anti-DNP mAb sensitized FLMCs or BMCMCs were washed with DMEM supplemented with 0.1% BSA (starvation medium), plated at 10^6^ cells per ml in the same medium, and then stimulated with DNP–HSA (2–200 ng ml^−1^) for 6 h. For the time course experiments, IgE anti-DNP mAb sensitized FLMCs were stimulated with DNP–HSA (2 or 20 ng ml^−1^) for 6, 12 and 20 h, respectively, in the combined presence of the following protease inhibitors: soybean trypsin inhibitor (SBTI; 100 μg ml^−1^, Sigma-Aldrich), potato carboxypeptidase inhibitor (PCI; 50 μg ml^−1^, Sigma-Aldrich) and chymostatin (60 μg ml^−1^, Sigma-Aldrich). Supernatants were collected for measurement of mIL-6, mTNF (BD Bioscience), mIL-13, mCCL2 (eBioscience) and mCCL3 (R&D Systems) protein levels by ELISA according to the manufacturer's instructions. The lower limits of detection were as follows: mIL-6=15.6 pg ml^−1^; mTNF=15.6 pg ml^−1^; mIL-13=4 pg ml^−1^; mCCL2=15 pg ml^−1^; and mCCL3=0.8 pg ml^−1^.

### Adoptive transfer of FLMCs or BMCMCs into mast cell-deficient mice

For mast cell engraftment studies, FLMCs or BMCMCs derived from WT B6-*Nedd4-2*^+/+^ (WT FLMCs or WT BMCMCs) or B6-*Nedd4-2*^*−/−*^ (*Nedd4-2*^*−/−*^ FLMCs or *Nedd4-2*^*−/−*^ BMCMCs) or B6-*Ndfip1*^*−/−*^ (*Ndfip1*^*−/−*^ BMCMCs) mice were transferred by intra-dermal injection (i.d., two injections into each ear with 1 × 10^6^ cells in 25 μl DMEM per injection) into 4–6-week-old male *Cpa3-Cre; Mcl-1*^*fl/fl*^ or *Kit*^W-sh/W-sh^ or *Kit*^W/W-v^ mice. Passive cutaneous anaphylaxis experiments were initiated 4–6 weeks after i.d. transfer of mast cell populations.

### IgE-dependent passive cutaneous anaphylaxis

For PCA, mice from all groups were injected i.d. with 20 μl of IgE anti-DNP mAb (SPE-7 clone or H1-DNP-ɛ-26 clone in experiments where indicated) at a concentration of 5 μg ml^−1^ (that is, 100 ng dose) diluted in Hanks' MEM containing 0.47 g l^−1^ piperazine-*N,N*′ bis (2-ethane sulfonic acid) (HMEM-Pipes; Sigma-Aldrich) in one ear and equal volume of HMEM-Pipes vehicle in the other ear of each mouse tested. Sixteen hours after IgE sensitization, all mice were injected i.v. (retro-orbitally) with 2 mg ml^−1^ of DNP–HSA-specific antigen diluted in 100 μl of sterile 0.9% saline (that is, dose of 200 μg). Ear thickness was measured using a dial thickness gauge (model G-1A; Ozaki MFG. Co., Ltd) before (baseline) and at intervals after i.v. antigen challenge. For Evans blue dye extravasation experiments, the PCA reaction was elicited as cited above with the exception that DNP–HSA (200 μg in 100 μl of sterile 0.9% saline containing 1% Evans blue dye (Gurr-Searle Diagnostic) was administered i.v. (tail vein). Mice were euthanized 30 min after i.v. DNP–HSA injection and whole ear pinnae were obtained and weighed. To extract the Evans blue dye, the ear pinnae were diced into pieces (∼1–2 mm^2^) in an Eppendorf tube and incubated in 0.3 ml formamide at 55 °C overnight. Samples were then centrifuged at 16,200*g* for 10 min and 100 μl of supernatant quantified by absorption at 610 nm on a plate reader. Data expressed as OD_610_/ear weight (g).

### Histology and quantification of mast cell numbers

Ear pinna samples were processed and histology analysed according to our established protocols^1^,^2^,^31^,^56^. Briefly, ear pinnae were fixed in 10% buffered formalin, embedded with a cross-sectional orientation in paraffin. Ear sections (4 μm) were stained with 0.1% Toluidine Blue (pH 1.0) for the detection of mast cells (cytoplasmic granules appear purple) and mast cells counted in 6–9 consecutive fixed fields of 870 μm width using a 20 × microscope objective (200 × final magnification). The entire length of each ear pinna extending from the base to the tip (∼5.4–8.1 mm) was quantified using computer-generated image analysis (NIH Image J software, version 1.46^r^). Numbers of mast cells were expressed per horizontal ear cartilage field length (millimetre). For representative images of inflammatory cell infiltrates, cross-sections of ear pinna were stained with haematoxylin and eosin, scanned using a Hamamatzu Nanozoomer 2.0HT (SDR Scientific) and images captured with Aperio ImageScope (V11.1.2.752) software.

### Flow cytometric analysis of leukocytes in ear skin

Flow cytometric assessment of PMN cells (Gr-1^+^F4/80^−^) and macrophages (Gr-1^−^F4/80^+^ and Gr-1^+^F4/80^+^) in ear pinnae was performed according to our established protocols^1^,^2^. Briefly, individual ears i.d. injected with IgE anti-DNP mAb or HMEM-Pipes vehicle of each mouse (in each group) were split parallel to the cartilage into two halves, diced and incubated in RPMI plus 0.5 mg ml^−1^ of Liberase TL Research Grade (Roche) for 2 h at 37 °C. Single cell suspensions were obtained by using a 70 μm nylon cell strainer. The cells were incubated with anti-mouse CD16/CD32 mAb (Clone 93, 0.5 μg ml^−1^, eBioscience) on ice for 15 min and then incubated on ice for 30 min with the following cell surface markers: anti-mouse CD45.2 (104, 0.5 μg ml^−1^, BD Biosciences), anti-mouse F4/80 (BM8, 1 μg ml^−1^, eBioscience) and Gr-1 (Ly-6C RB6-8C5, 0.5 μg ml^−1^, BD Biosciences). Live cells as determined by Live/Dead Fixable Aqua Dead Cell Stain (Life Technologies) or Fixable Viability Stain 700 (BD Biosciences) were used for analyses. For Fig. 3c, data were collected on a Gallios flow cytometer (Beckman Coulter) and analysed using FCS express 4 (version 4.07.0014, *De Novo* software). For Fig. 7c, data were collected on a BD LSR Fortessa flow cytometer (BD Biosciences) and analysed using FCS express 4 (version 4.07.0014, *De Novo* software). Gates for subpopulations of cells were based on single colour stain of the cells to determine compensation and non-specific fluorescence. To calculate the number of live cells of a particular type recovered per ear (determined by gating on Live/Dead Aqua-negative or Fixable Viability Stain 700-negative cells), the following calculation was applied for each population quantified: live cells recovered per ear of PMNs or macrophages=(percentage gated of the total cell population in that group) × (total number of cells recovered from the ears).

### Measurement of cytokines in ear skin lysates

Ear skin lysates were prepared in PBS containing protease inhibitors as previously described^1^,^2^. TNF, IL-6, CCL2 and CCL3 protein levels in the supernatants were measured by ELISA (eBioscience), according to the manufacturer's instructions, and data obtained for each group were expressed as median±range (box and whiskers) pictogram/milligram protein. Total protein levels in the supernatants were measure by a Bio-Rad D_c_ protein assay, according to the manufacturer's instructions (Bio-Rad Laboratories).

### Immunoblotting and immunoprecipitation

FLMCs were incubated with IgE anti-DNP mAb (SPE-7 clone, 2 μg ml^−1^) for 16 h at 37 °C in a CO_2_ incubator, centrifuged at 180*g* for 5 min, resuspended in Tyrode's buffer, centrifuged again, and then resuspended with Tyrode's buffer (2 × 10^6^ cells per sample for analysis of total cell lysates or 20 × 10^6^ cells per samples for immunoprecipitation). FLMCs were activated with 20 ng ml^−1^ DNP–HSA-specific antigen at various intervals up to 60 min at 37 °C. The reaction was quenched by the addition of 500 μl ice-cold buffer, followed immediately by centrifugation at 180 *g* for 5 min at 4 °C. Cells were then lysed in 50 μl ice-cold lysis buffer containing 50 mM Tris-base, 100 mM NaCl, 5 mM EDTA, 10 mM Na_4_P_2_O_7_, 1% Triton X-100, 1 mM PMSF, 2 mM NaF, 1 × complete protease inhibitors cocktail (Roche). Total cell lysates were separated with SDS–polyacrylamide gel electrophoresis (SDS–PAGE) and transferred to nitrocellulose membranes. Membranes were blocked in 5% nonfat dry milk in Tris-buffered saline that contained 0.1% Tween buffer; they were then probed with antibodies raised in rabbit against the phosphorylated form of ERK1/2 (Thr202/Tyr204, #9101), p38 (Thr180/Tyr182, #4511), JNK1/2 (Thr183/Tyr185, #4668), NF-κB-p65 (Ser536, #3033), Syk (Tyr525/526, #2710, only detects p-Syk and does not cross-react with total Syk), Lyn (Tyr507, #2731), and LAT1 (Tyr191, #07–278) at a dilution of 1:1,000 (except pLAT at 1:500) overnight at 4 °C. All antibodies with the exception of p-LAT1 (Millipore) were obtained from Cell Signaling Technology. Membranes were then probed with horseradish peroxidase (HRP)-conjugated antibody against rabbit IgG (1:3,000 dilution, #7074), and bands visualized with ECL reagent (Amersham, GE Healthcare Life Sciences) with a LAS4000 imaging system (Fujifilm). Membranes were then stripped and re-probed with antibodies against the total form of these proteins (ERK1/2 #4695, p38 #9212, JNK1/2 #9258, NF-κB-p65 #4767, Syk #2712, Lyn #2796, LAT1 #9166). The band intensity was quantified using Scion Image software (Scion Corporation). Original immunoblots are shown in Supplementary Fig. 9.

For immunoblots of mast cell-Nedd4-2 expression, FLMCs or BMCMCs (2 × 10^6^ cells per sample) were lysed in ice-cold lysis buffer, membranes prepared as indicated above and then probed with affinity purified rabbit anti-Nedd4-2 (ref. 57) and anti-β-actin (#4967, Cell Signaling Technology), antibodies, both diluted 1:1,000. HRP-conjugated rabbit IgG secondary antibody was then applied and bands visualized as detailed above.

For the IL-3-induced signalling study, FLMCs (2 × 10^6^ cells per sample) were incubated in starvation medium (DMEM supplemented with 0.1% BSA) for 6 h and then stimulated with recombinant mouse IL-3 (4 ng ml^−1^, Shenandoah Biotechnology) for the indicated times at 37 °C. The reaction was quenched as outlined above, cells prepared in ice cold lysis buffer, lysates transferred to nitrocellulose membranes, and these then probed with antibodies against p-STAT5 (1:1,000 dilution, Tyr694, #611964, BD Bioscience) and p-ERK1/2 (1:1,000 dilution, Thr202/Tyr204). HRP-conjugated mouse (for p-STAT5) or rabbit IgG (for p-ERK1/2) secondary antibodies were used (1:3,000 dilution) and membranes stripped then re-probed with total-ERK1/2 (1:1,000 dilution) and total-STAT5 (1:1,000 dilution, #610191, BD Bioscience) antibodies as controls.

For immunoprecipitation studies, FLMCs (20 × 10^6^ cells per sample) were sensitized with IgE anti-DNP mAb, activated with DNP–HSA and prepared as indicated above. Following lysis in 500 μl ice-cold lysis buffer, the lysates were first pre-cleared with protein A Sepharose (Amersham Biosciences), followed by incubation with indicated Abs (Syk, p-Syk, or affinity purified rabbit anti-mouse Ndfip1 (generated by S. Kumar) for 1 h (1 μl of antibody in 60 μl Protein A Sepharose per sample), before immunoprecipitation with Protein A Sepharose for another hour. Immunoprecipitated samples were washed with lysis buffer four times (centrifugation at 180*g* for 5 min at 4 °C) before immunoblot analysis with Nedd4-2, Syk, p-Syk or Ndfip1 antibodies (all 1:1,000 dilution), and HRP-conjugated rabbit IgG secondary antibody (1:3,000 dilution). For immunoblots associated with immunoprecipitated Ndfip1, Protein A HRP-conjugated secondary antibody (1:10,000 dilution, Thermo Scientific) was used to avoid visualization of the IgG light chain).

### Affinity purification of ubiquitinated proteins using TUBEs

After IgE sensitization for 16 h, cells were incubated with MG132 (25 μM; Merck) and chloroquine (50 μM; Sigma-Aldrich) for 2 h before DNP–HSA-mediated activation. FLMCs or BMCMCs (20 × 10^6^ cells per sample) were lysed in 500 μl ice-cold lysis buffer. Cell lysates were then rotated at 4 °C with 20 μl Agarose-Tandem Ubiquitin Binding Entities (TUBEs) (Lifesensors) for 2 h. Beads were collected, washed (three times) in cold tris-buffered saline with Tween-20 (TBS-T) and samples then subjected to SDS–PAGE, followed by immunoblot with Syk, p-Syk or p-Lyn antibodies (all 1:1,000 dilution).

### Calcium mobilization study

IgE anti-DNP (2 μg ml^−1^; SPE7 clone) sensitized FLMCs or BMCMCs were administered with 5 μM Fluo-3 AM (Life Technologies) in Tyrodes buffer for 30 min at 37 °C and then placed in a 15μ-slide Microscopy Chamber (ibidi GmbH). Fluorometric measurements were commenced when >95% of the cells had settled to the base of the chamber. A Biorad Radiance 2000 Confocal system mounted on an inverted IX81 Olympus microscope, equipped with a 20 × water-immersion objective (numerical aperture (NA) 0.5) and fluorescence intensity excited at 488 nm and emitted at 520 nm was measured at 5 s intervals for 5 min after DNP–HSA (10 ng ml^−1^) stimulation. Changes in intracellular Ca^2+^ [Ca^2+^]_i_ were expressed as *F*1/*F*0 ratios where *F*1 and *F*0 was the fluorescence intensity at a specific time and at the initiation of image recording. For each independent experiment using paired WT and *Nedd4-2*^*−/−*^ FLMCs or BMCMCs, two–three replicates were performed to acquire information on 200–300 cells.

### RNA extraction and real-time PCR

FLMCs (2 × 10^6^ cells) were sensitized with IgE anti-DNP mAb (2 μg ml^−1^, SPE-7 clone) for 16 h, then lysed in 500 μl TRIzol reagent (Life Technologies) from which RNA was extracted according to the manufacturer's instructions. For mRNA analysis, 1 μg of RNA was used for complementary DNA (cDNA) synthesis using the QuantiTect reverse transcription kit (QIAGEN). Conventional PCR was performed using GoTAQ green master mix reagent (Promega) on S1000 Thermal Cycler (BioRad). PCR assays were performed for 30 cycles (95° C for 30 s, 57° C for 30 s, and 72 °C for 60 s). PCR products were run on a 2% agarose gel and visualized using a GelDot-IT TS Imaging System (UVP). The following oligonucleotide sequences were used:


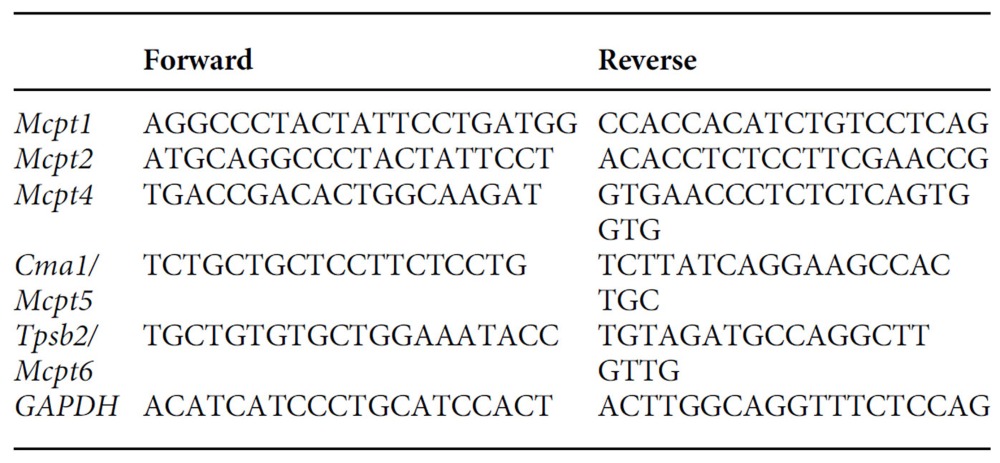


### Statistical analysis

Prism software version 5.01 (GraphPad Software) was used for statistical analyses. A two-way analysis of variance (ANOVA) with Bonferroni post test for repeated measures was used to assess differences in ear swelling between groups of mice over the course of the PCA reactions or to compare differences in mediator release in response to increasing concentrations of DNP–HSA specific antigen between WT and *Nedd4-2*^*−/−*^ FLMCs or BMCMCs, or *Ndfip1*^−/−^ BMCMCs. Where specified, a one-way ANOVA with Dunnett's or Bonferroni post test for comparison between multiple groups or an unpaired Student's *t*-test for comparison between two groups were used. A *P* value of <0.05 was considered statistically significant. Data are presented as mean±s.e.m., unless otherwise stated.

### Data availability

All relevant data are available within the article and its Supplementary Files or available from from the authors upon request.

## Additional information

**How to cite this article:** Yip, K. H. *et al*. The Nedd4-2/Ndfip1 axis is a negative regulator of IgE-mediated mast cell activation. *Nat. Commun.* **7,** 13198 doi: 10.1038/ncomms13198 (2016).

## Supplementary Material

Supplementary Information Supplementary Figures 1–9
