# Supplementary material for: The Nedd4-2/Ndfip1 axis is a negative regulator of IgE-mediated mast cell activation
Source: Nat Commun. 2016 Oct 27;7:13198. doi: 10.1038/ncomms13198 (PMC5095291; doi:10.1038/ncomms13198)
Supplement: Supplementary Information — Supplementary Figures 1–9 [file ncomms13198-s1.pdf]

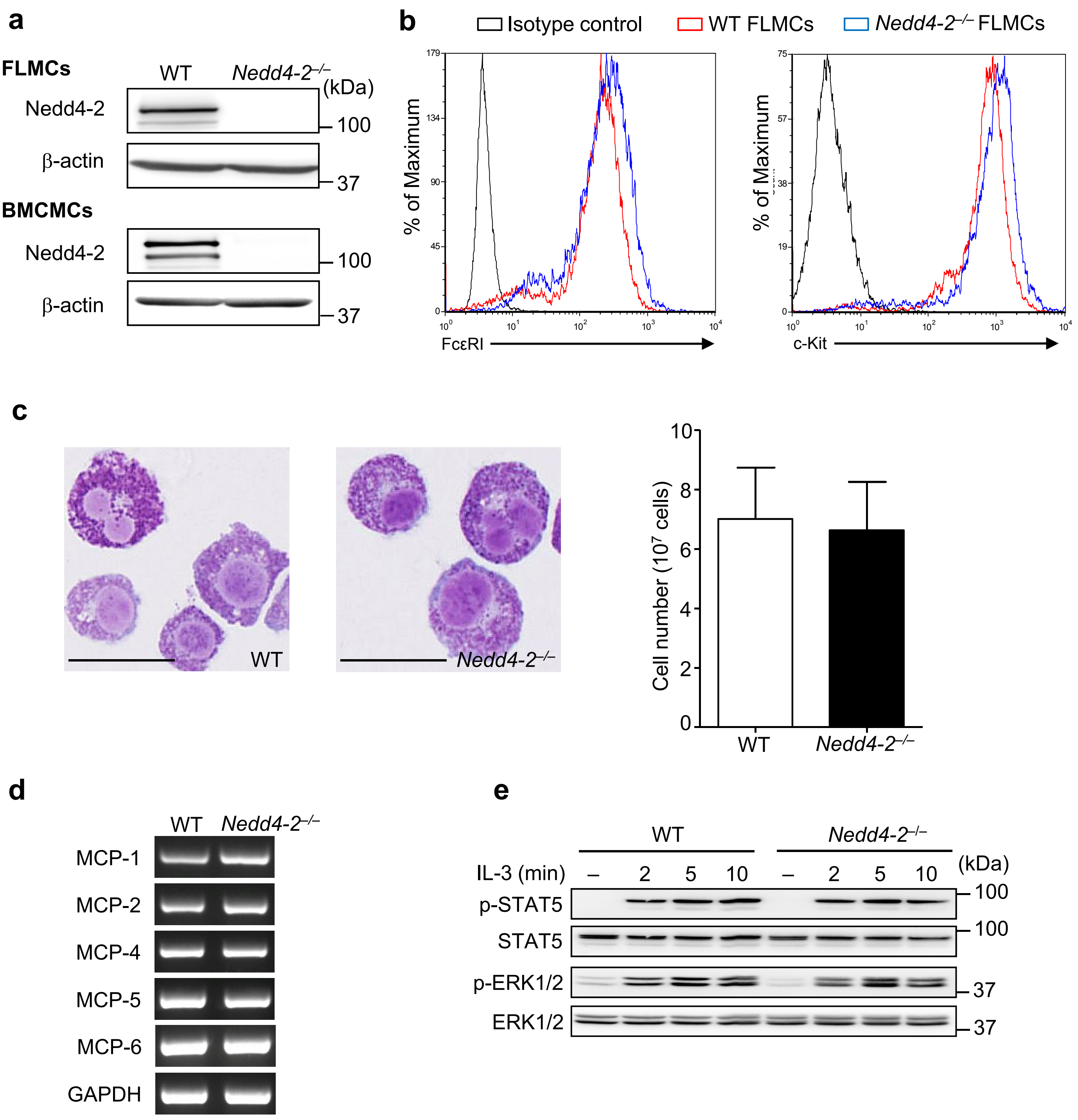

Supplementary Figure 1. Nedd4-2 does not affect IL-3–dependent mast cell growth and development.

(a) Immunoblot analysis of Nedd4-2 expression in WT or *Nedd4-2*$^{-/-}$ FLMCs. **(b)** Flow cytometric analysis of FcεRI and c-Kit expression in WT or *Nedd4-2*$^{-/-}$ FLMCs at 6 week of culture. **(c)** May-Grűnwald Giemsa staining of WT and *Nedd4-2*$^{-/-}$ FLMCs (left panel; Bar: 25 μm) and total mast cell numbers obtained at the end of 6 week culture (right panel). **(d)** mRNA levels of mast cell proteases in WT and *Nedd4-2*$^{-/-}$ FLMCs after sensitization with IgE anti-DNP (SPE-7, 2 μg/ml). **(e)** FLMCs starved in 0.1% BSA DMEM for 6 h and then stimulated with recombinant mouse IL-3 (4 ng/ml) for the indicated times at 37 °C. Immunoblot analysis of phosphorylated STAT5 or ERK1/2 in total cell lysates. Total-STAT5 and total- ERK1/2 were used as a loading control. Data (**a**,**b**,**d**) are representative of, or pooled (**c**, mean ± s.e.m.) from, nine independent paired WT and *Nedd4-2*$^{-/-}$ FLMC cultures. Panel **e** is representative of the three independent experiments performed, each of which gave similar results.

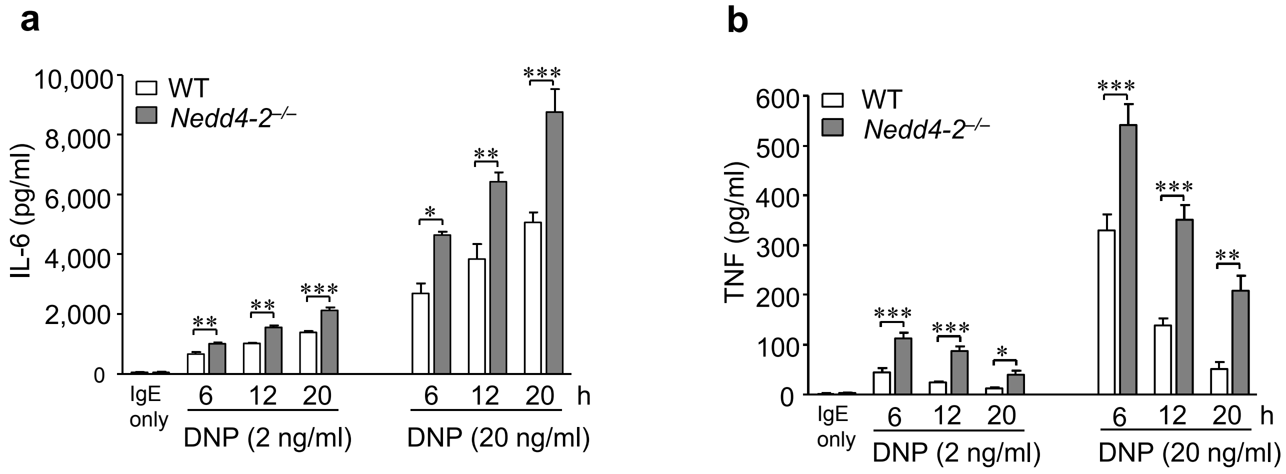

Supplementary Figure 2. **Loss of Nedd4-2 results in sustained cytokine production in IgE-activated mast cells.**

Release of (**a**) IL-6 and (**b**) TNF in IgE (SPE-7, 2 μg/ml) sensitized WT and *Nedd4-2*$^{-/-}$ FLMCs stimulated with 2 or 20 ng/ml DNP-HSA for 6, 12 and 20 h. Data (mean ± s.e.m.) are pooled from the three independent experiments performed, each of which gave similar results. *$p < 0.05$, **$p < 0.01$, ***$p < 0.001$ for indicated comparisons (two-way ANOVA with Bonferroni post-test).

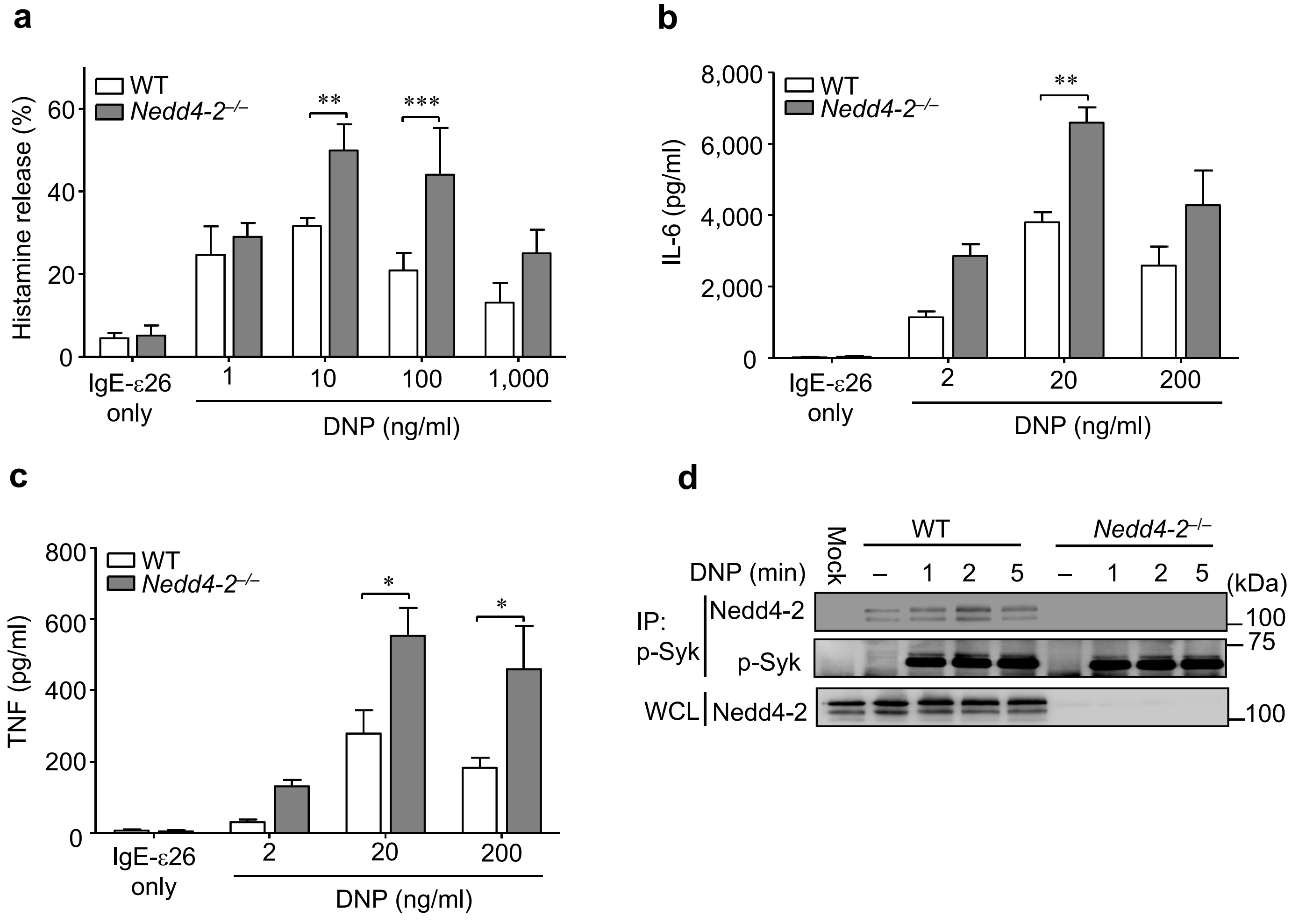

Supplementary Figure 3. Enhanced mediator release by *Nedd4-2*$^{-/-}$ mast cells is observed with mast cell sensitized with H1-DNP-ε26 anti-DNP IgE.

Release of **(a)** Histamine (30 min), **(b)** IL-6 (6 h) and **(c)** TNF (6 h) by WT or *Nedd4-2*$^{-/-}$ FLMCs sensitized with anti-DNP IgE (H1-DNP-ε26, 2 µg/ml) overnight and then stimulated with indicated concentrations of DNP-HSA. Data (mean $\pm$ s.e.m.) are pooled from the three independent experiments performed, each of which gave similar results. **p* < 0.05, ***p* < 0.01, ****p* < 0.001 for indicated comparisons (two-way ANOVA with Bonferroni post-test). (**d**) Nedd4-2 interacts with p-Syk in response to H1-DNP-ε26 IgE (2 µg/ml) + DNP-HSA (20 ng/ml) activation in WT but not in *Nedd4-2*$^{-/-}$ FLMCs. Immunoblot analysis of Nedd4-2 immunoprecipitated (IP) with anti-p-Syk in lysates of WT and *Nedd4-2*$^{-/-}$ FLMCs stimulated with DNP-HSA for the indicated times.

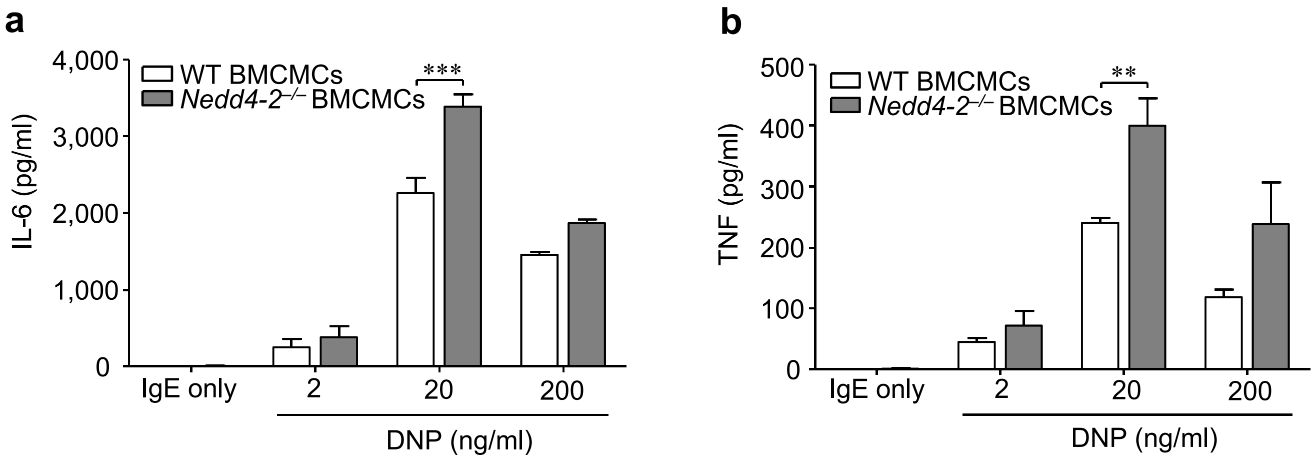

Supplementary Figure 4. Enhanced IgE-dependent cytokine production by *Nedd4-2*$^{-/-}$ BMCMCs.

WT and *Nedd4-2*$^{-/-}$ BMCMCs were sensitized with IgE anti-DNP antibody (SPE-7, 2 μg/ml) for 16 h, then stimulated for 6 h with indicated concentrations of DNP-HSA and levels of secreted **(a)** IL-6 and **(b)** TNF were measured. Data (mean $\pm$ s.e.m.) are pooled from the three independent experiments performed, each of which gave similar results. $^{**}p < 0.01$, or $^{***}p < 0.001$ for indicated comparisons (two-way ANOVA with Bonferroni post-test).

.

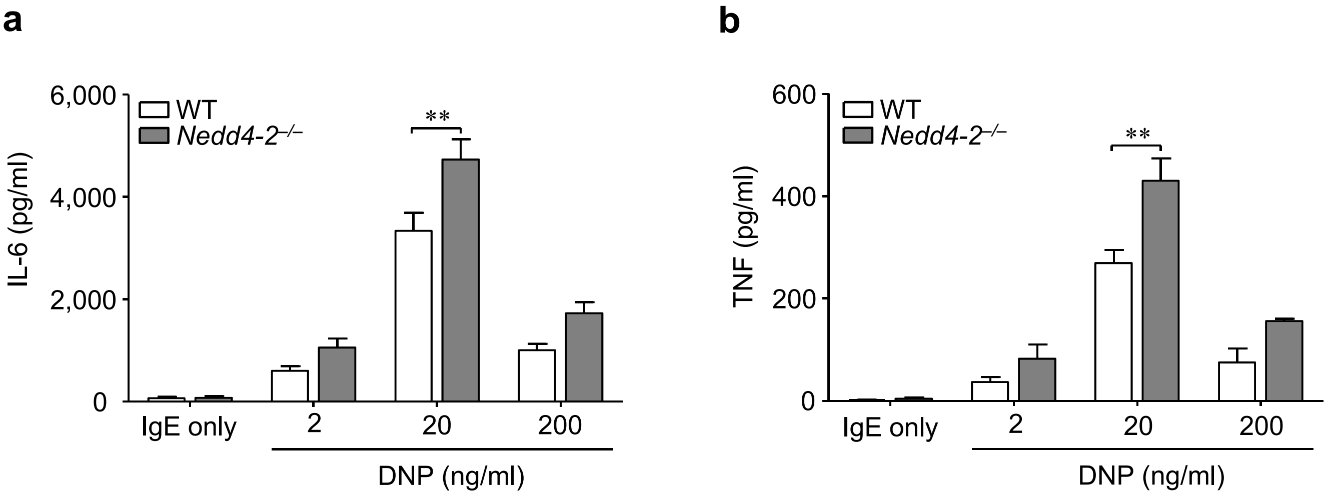

Supplementary Figure 5. Enhanced IgE-dependent cytokine production by *Nedd4-2*$^{-/-}$ FLMCs cultured in IL-3 and stem cell factor.

WT and *Nedd4-2*$^{-/-}$ FLMCs generated in IL-3 (3 ng/ml) and SCF (50 ng/ml) medium throughout development of fetal liver progenitor cells into mature mast cells, were sensitized with anti-DNP IgE (SPE-7, 2 μg/ml) overnight and then stimulated for 6 h with indicated concentrations of DNP-HSA and levels of secreted **(a)** IL-6 and **(b)** TNF were measured. Data (mean ± s.e.m.) are pooled from the three independent experiments performed, each of which gave similar results. $^{**}p < 0.01$ for indicated comparisons (two-way ANOVA with Bonferroni post-test).

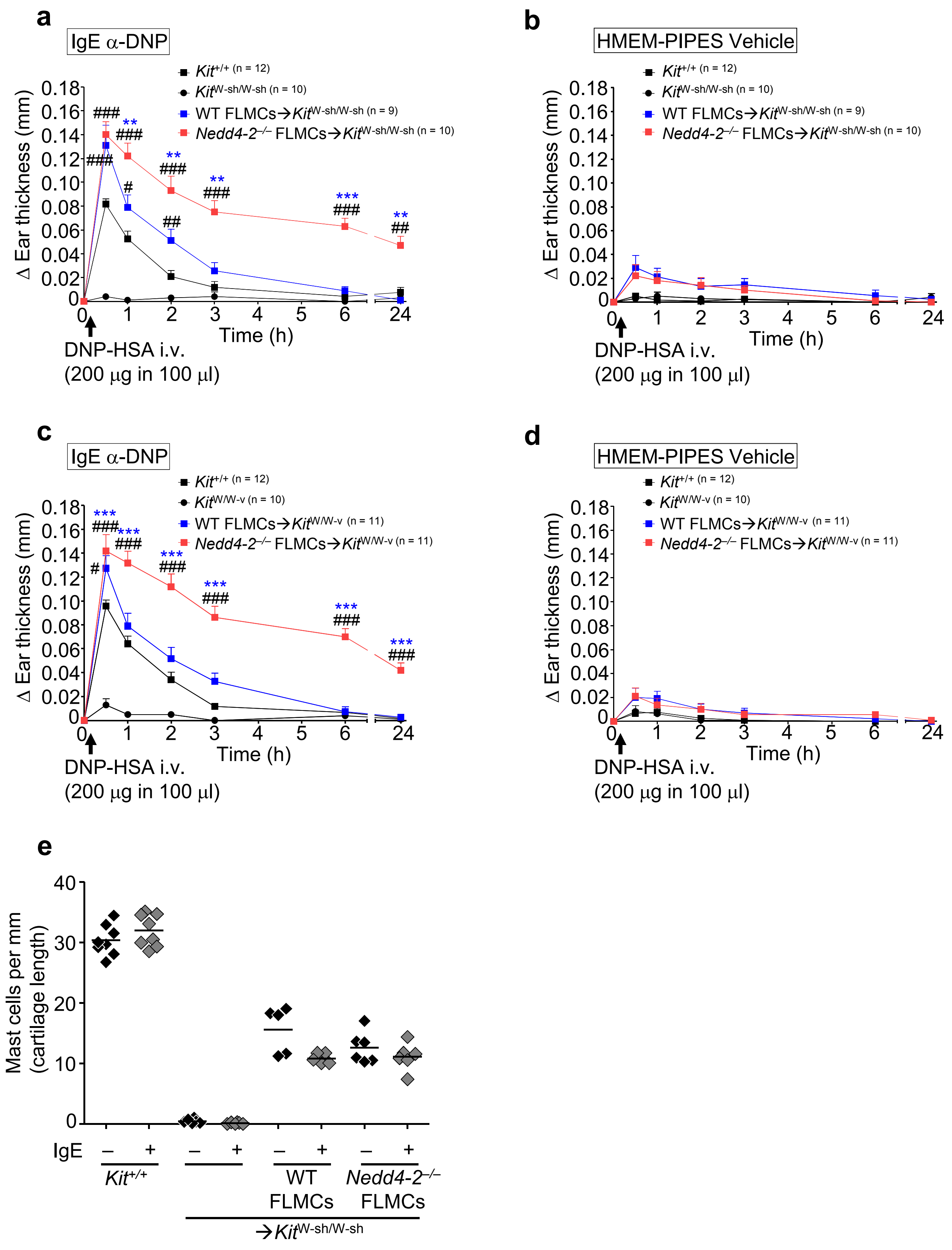
a
IgE α-DNP
Kit+/+ (n = 12)
KitW-sh/W-sh (n = 10)
WT FLMCs→KitW-sh/W-sh (n = 9)
Nedd4-2–/– FLMCs→KitW-sh/W-sh (n = 10)
Δ Ear thickness (mm)
Time (h)
DNP-HSA i.v.
(200 μg in 100 μl)
b
HMEM-PIPES Vehicle
Kit+/+ (n = 12)
KitW-sh/W-sh (n = 10)
WT FLMCs→KitW-sh/W-sh (n = 9)
Nedd4-2–/– FLMCs→KitW-sh/W-sh (n = 10)
Δ Ear thickness (mm)
Time (h)
DNP-HSA i.v.
(200 μg in 100 μl)
c
IgE α-DNP
Kit+/+ (n = 12)
KitW/W-v (n = 10)
WT FLMCs→KitW/W-v (n = 11)
Nedd4-2–/– FLMCs→KitW/W-v (n = 11)
Δ Ear thickness (mm)
Time (h)
DNP-HSA i.v.
(200 μg in 100 μl)
d
HMEM-PIPES Vehicle
Kit+/+ (n = 12)
KitW/W-v (n = 10)
WT FLMCs→KitW/W-v (n = 11)
Nedd4-2–/– FLMCs→KitW/W-v (n = 11)
Δ Ear thickness (mm)
Time (h)
DNP-HSA i.v.
(200 μg in 100 μl)
e
Mast cells per mm
(cartilage length)
IgE – + – + – + – +
Kit+/+
WT FLMCs
Nedd4-2–/– FLMCs
→KitW-sh/W-sh

Supplementary Figure 6. Mast cell-Nedd4-2 restrains IgE-mediated passive cutaneous anaphylaxis (PCA).

Changes (Δ) in ear thickness 0 – 24 h after *i.v.* injection of DNP-HSA (200 μg in 100 μl) into mice, with DNP-HSA given 16 h after *i.d.* injection of anti-DNP IgE (SPE-7, 100 ng) in the right ear pinna (**a**, **c**) and equal volume of HMEM-Pipes vehicle in the left ear pinna (**b**, **d**) of (**a, b**) C57BL/6J-$Kit^{+/+}$ mice (■), mast cell-deficient $Kit^{W\text{-}sh/W\text{-}sh}$ mice (●), and mast cell-deficient mice engrafted *i.d.* with WT FLMCs (WT FLMCs→$Kit^{W\text{-}sh/W\text{-}sh}$, ■) or $Nedd4\text{-}2^{-/-}$ FLMCs ($Nedd4\text{-}2^{-/-}$ FLMCs→$Kit^{W\text{-}sh/W\text{-}sh}$, ■), and (**c**, **d**) WBB6F1-$Kit^{+/+}$ mice (■), mast cell-deficient $Kit^{W/W\text{-}v}$ mice (●), WT FLMCs→$Kit^{W/W\text{-}v}$ mice (■) or $Nedd4\text{-}2^{-/-}$ FLMCs→$Kit^{W/W\text{-}v}$ mice (■). Data (mean ± s.e.m.) are pooled from the three independent experiments performed, each of which gave similar results, each with 3 to 5 mice per group. $^{**}p < 0.01$, $^{***}p < 0.001$ for comparisons of WT FLMCs versus $Nedd4\text{-}2^{-/-}$ FLMCs→mast cell-deficient mice. $^{\#}p < 0.05$, $^{\#\#}p < 0.01$, $^{\#\#\#}p < 0.001$ for comparisons of WT mice versus WT FLMCs or $Nedd4\text{-}2^{-/-}$ FLMCs→mast cell-deficient mice (two-way ANOVA with Bonferroni post-test). (**e**) Dermal mast cell numbers in ear pinnae of mice at the completion of two of the PCA experiments (i.e. at 24 h after injction of DNP-HSA) outlined in (**a**, **b**).

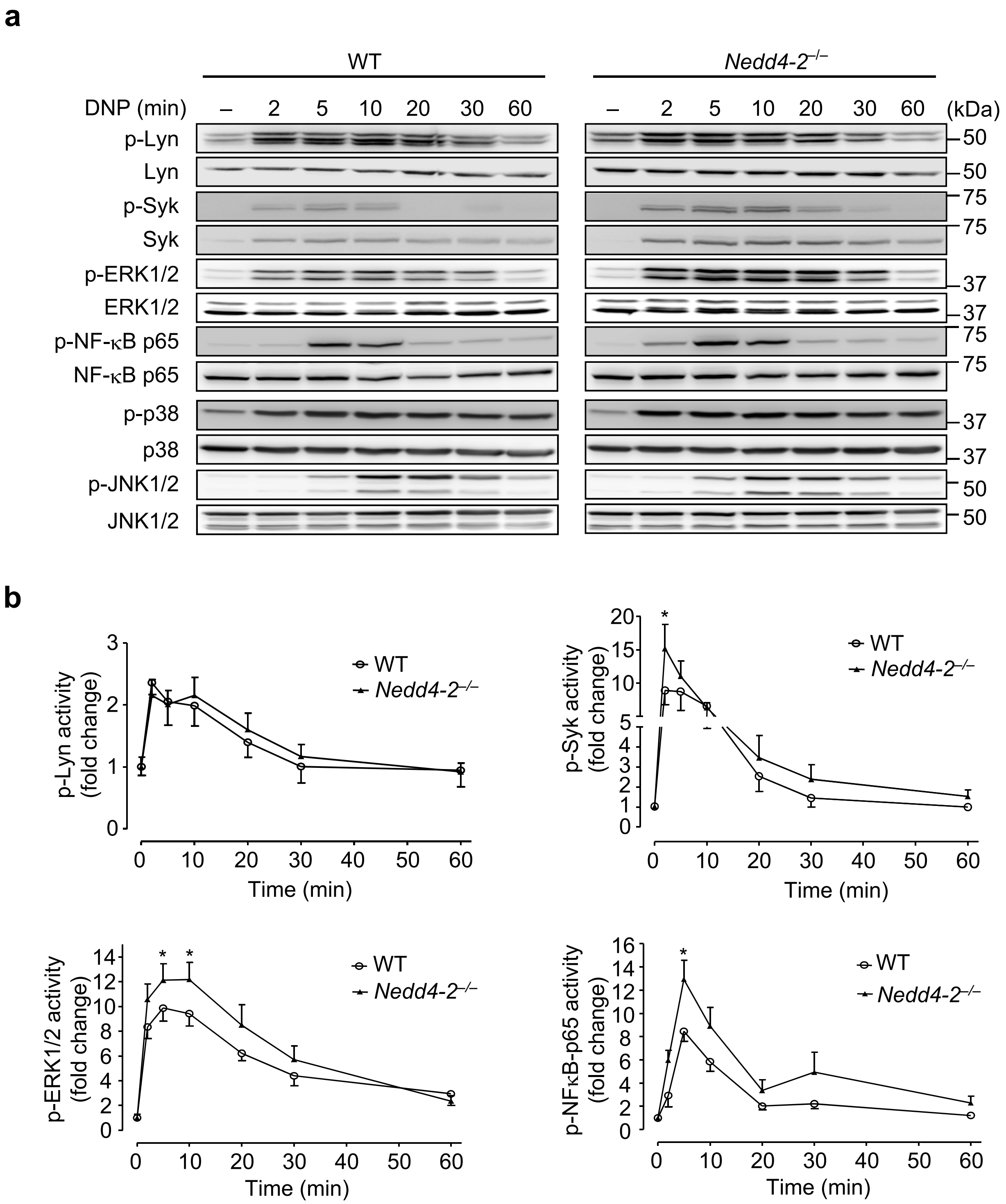

Supplementary Figure 7. Enhanced IgE-mediated signaling activity in Nedd4-2 deficient mast cells.

(a) Immunoblot analysis of phosphorylated (p-) and total signaling Lyn, Syk, LAT1, ERK1/2, NF-κB-p65, p38 and JNK1/2 proteins in whole cell lysates prepared from IgE anti-DNP (SPE-7, 2 μg/ml) sensitized WT or *Nedd4-2*$^{-/-}$ FLMCs stimulated with DNP-HSA (20 ng/ml) for the indicated time points. (**b**) Quantification of immunoblot band intensities measured by scion image software and data normalized with total form of the corresponding protein and then to the un-stimulated control. Data (mean ± s.e.m.), *$p < 0.05$ for indicated comparisons (two-way ANOVA with Bonferroni post-test). Data are representative of the two to six (**a**) or six (**b**) independent experiments performed, each of which gave similar results.

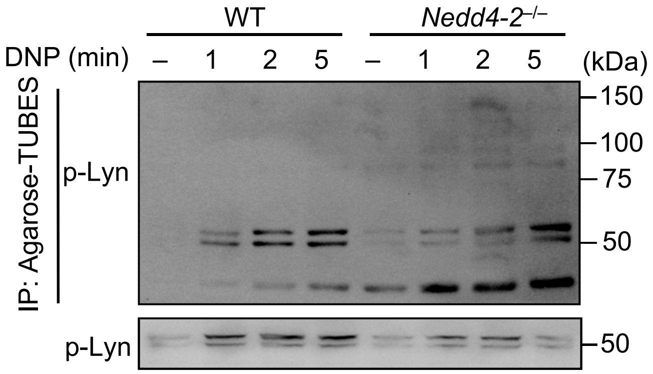

Supplementary Figure 8. **Nedd4-2 is not essential for p-Lyn ubiquitination in mast cells.**

Ubiquitylation of p-Lyn using Agarose-Tandem Ubiquitin Binding Entities in cell extracts from IgE anti-DNP (SPE-7, 2 µg/ml) sensitized WT and *Nedd4-2*$^{-/-}$ FLMCs treated with MG132 (25 µM) and chloroquine (50 µM) for 2 h prior to stimulation with DNP-HSA (20 ng/ml) for the indicated times. Data are representative of the three independent experiments performed, each of which gave similar results.

Supplementary Figure 9.

Note: The following pages (Supplementary Figure 9) show original images of immunoblots used in various main and supplementary figures. The legend of this figure follows the images.

Figure 4a

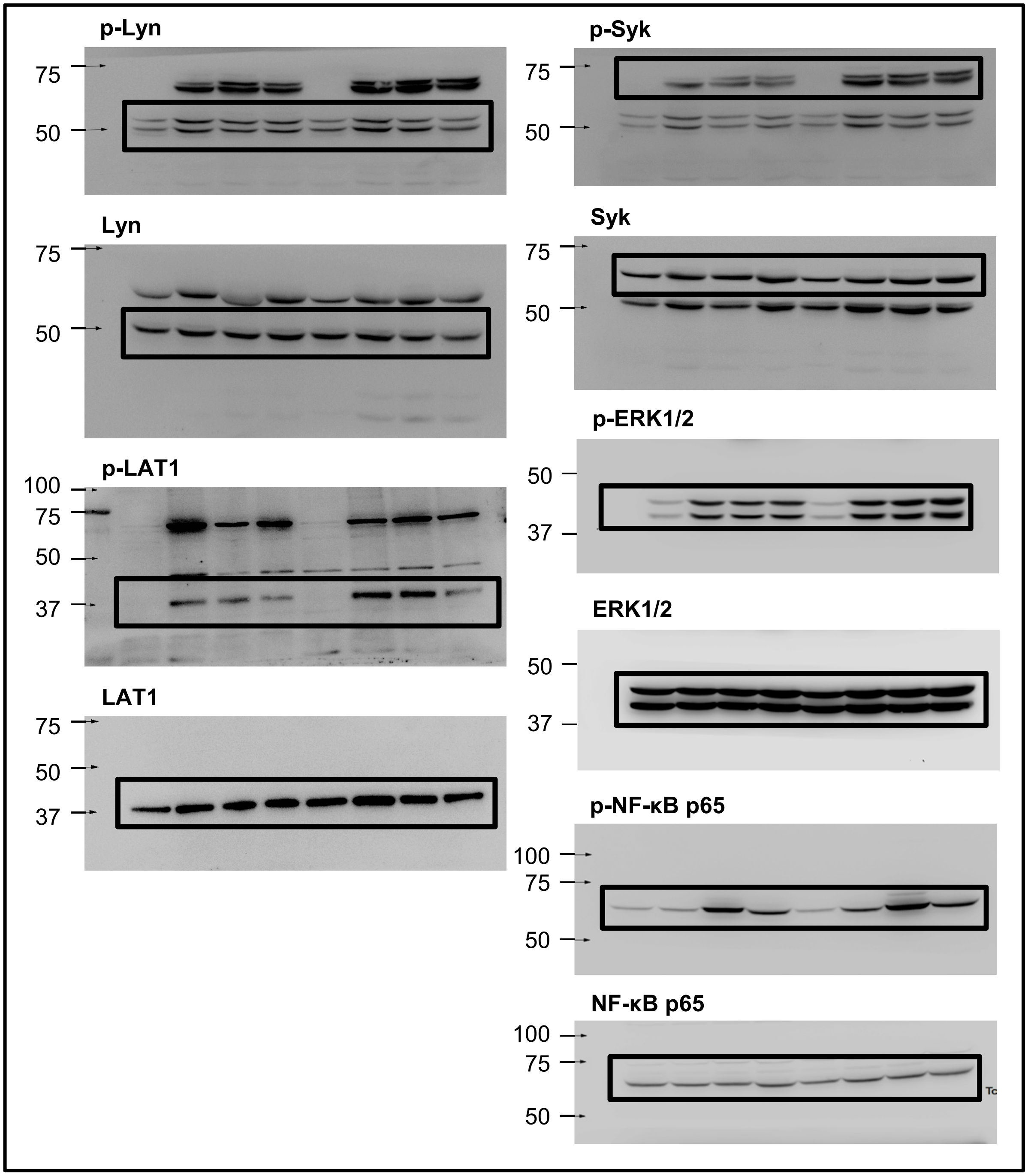

Supplementary Figure 9.

Figure 4b

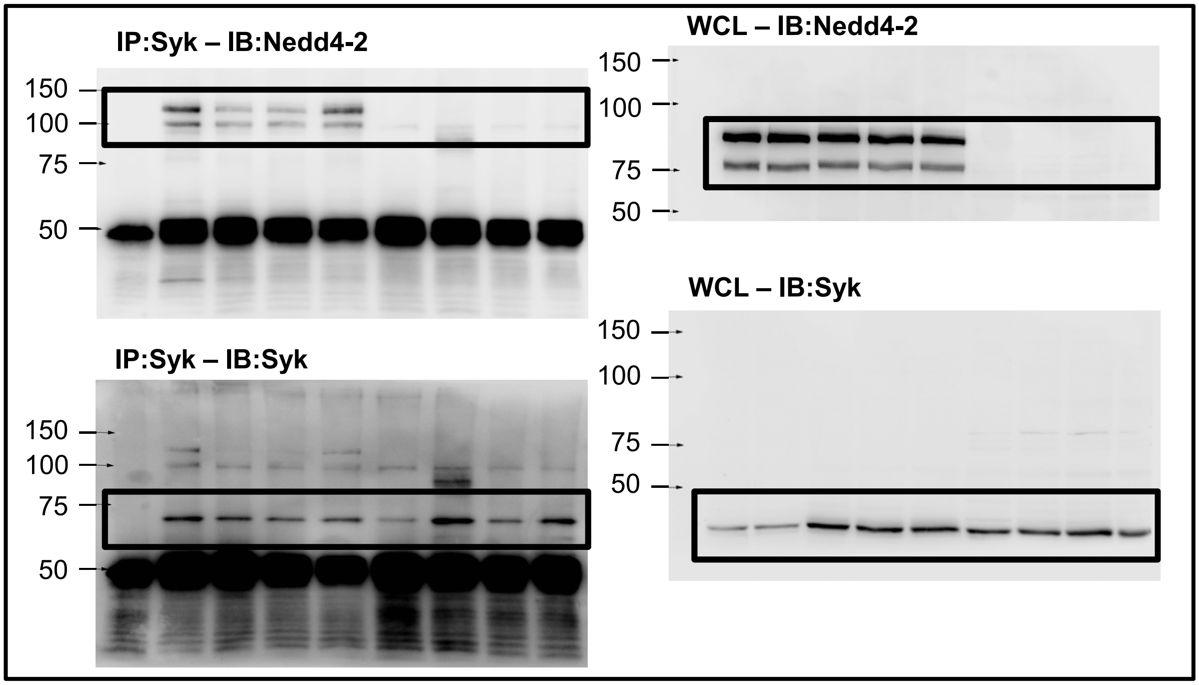

Figure 4c

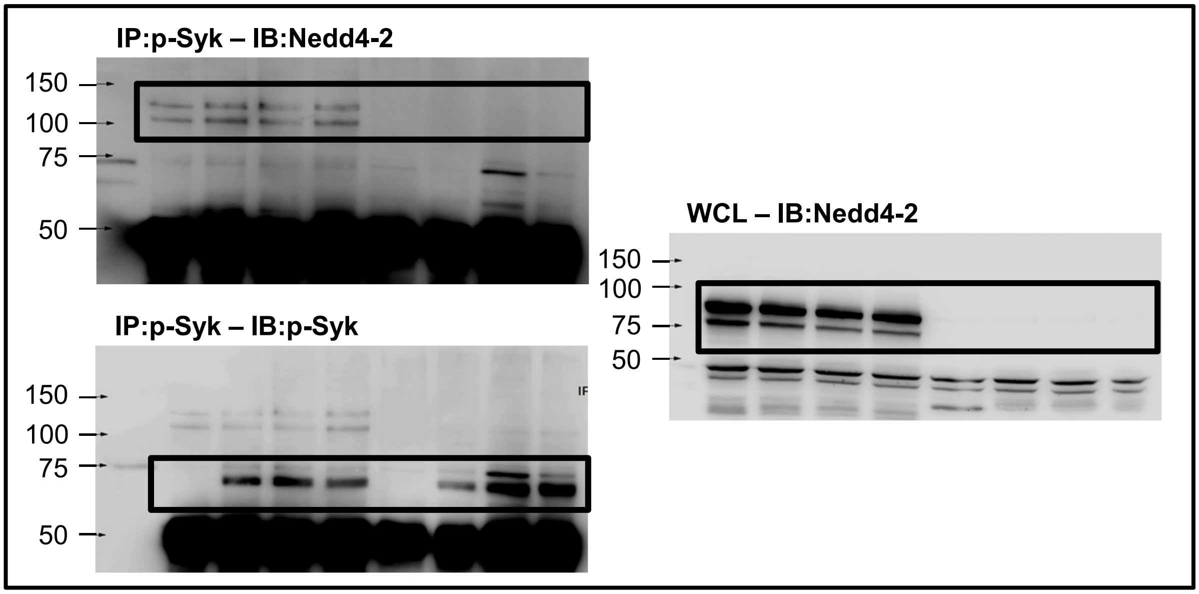

Figure 4d

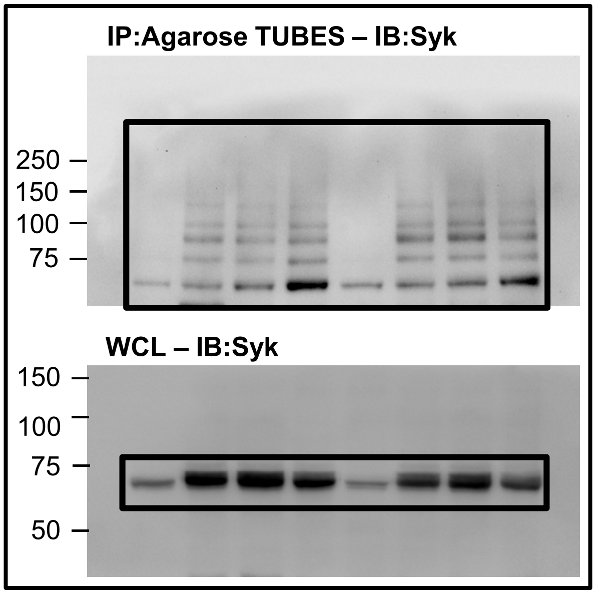

Figure 4e

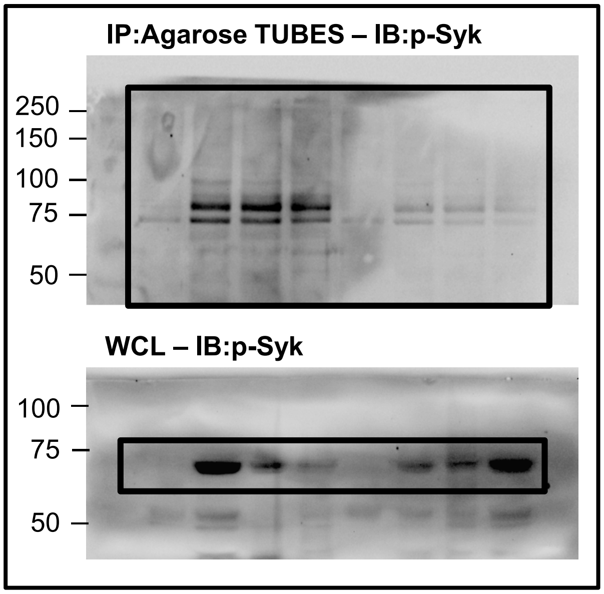

Supplementary Figure 9.

Figure 5a

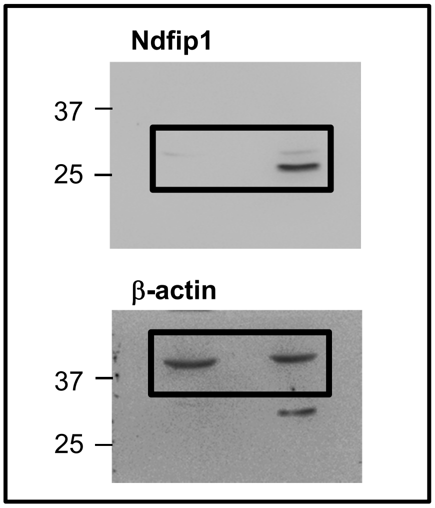

Figure 6a

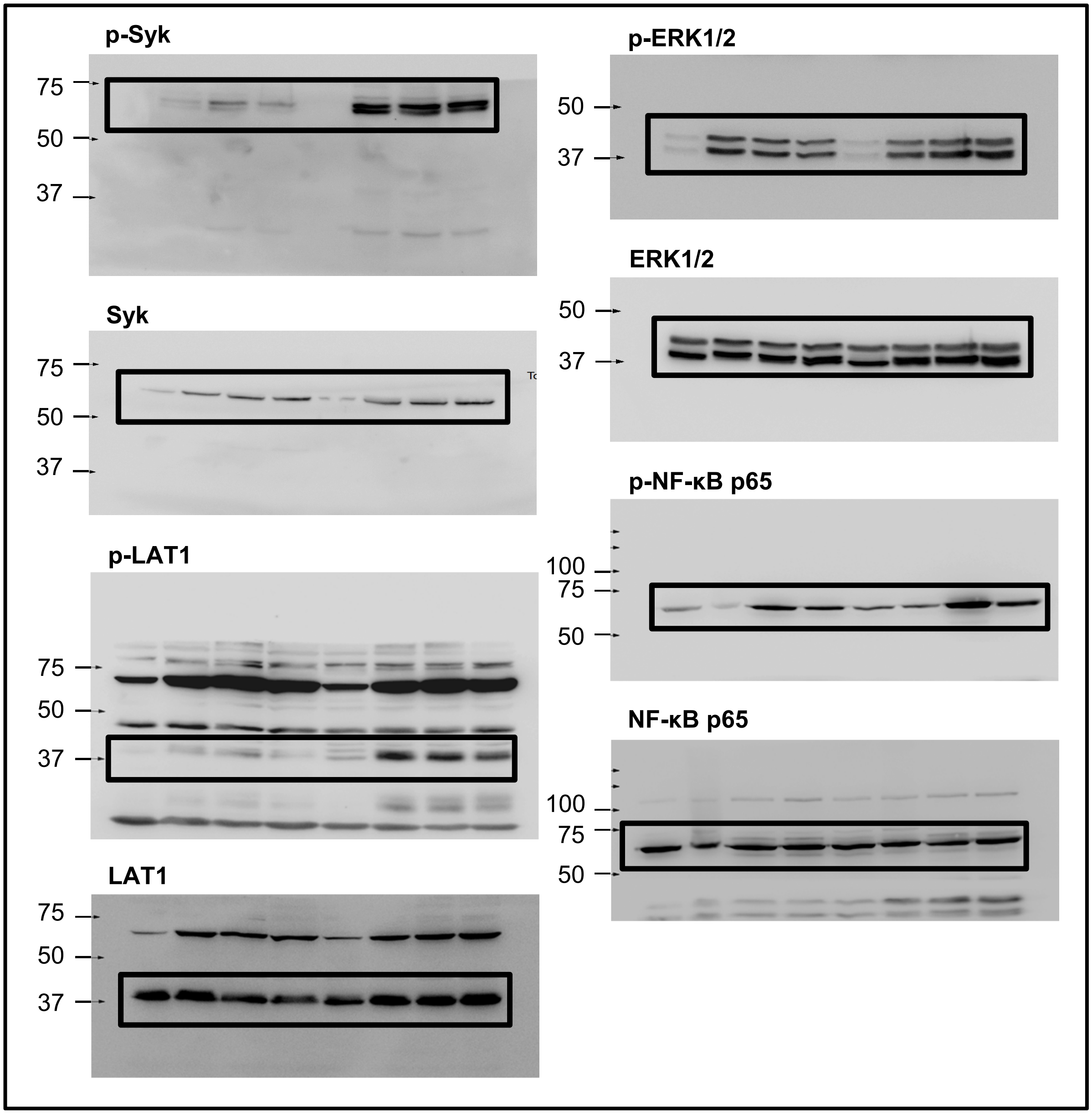

Supplementary Figure 9.

Figure 6c

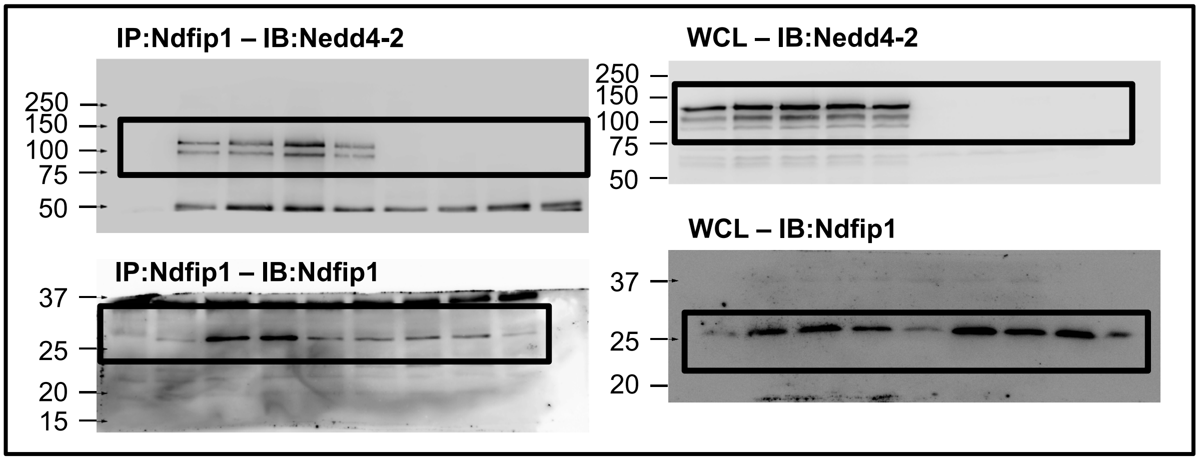

Figure 6d

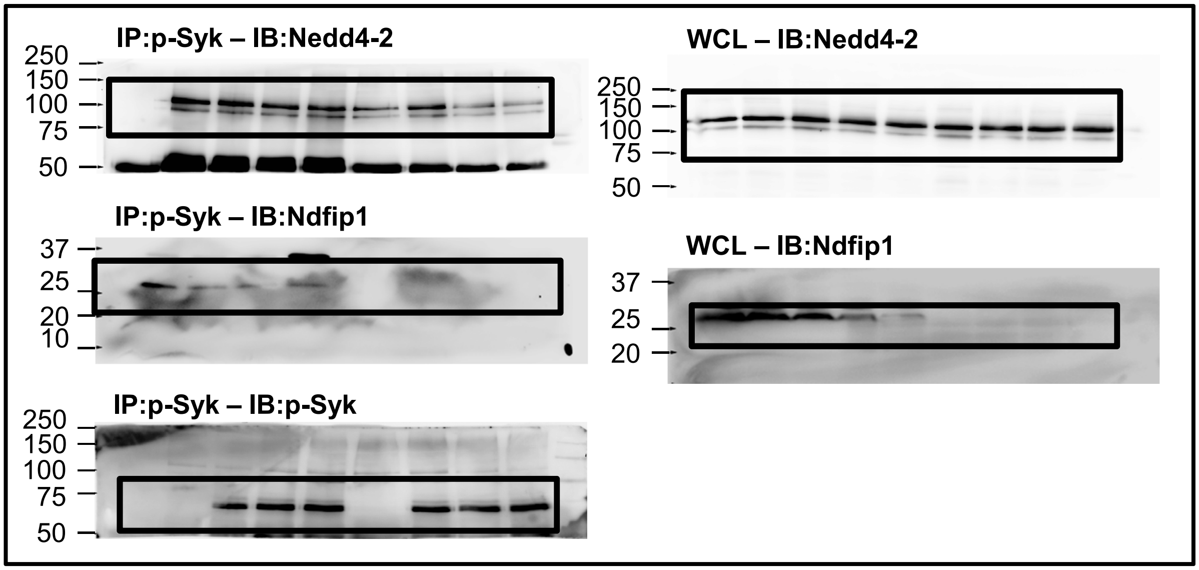

Figure 6e

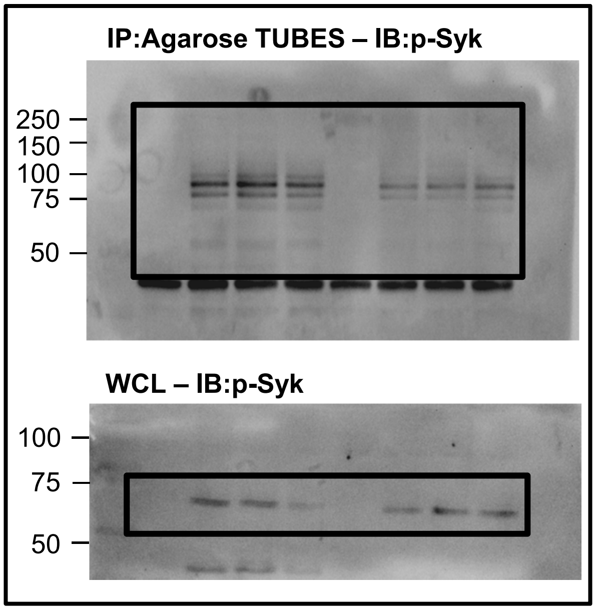

Figure 6f

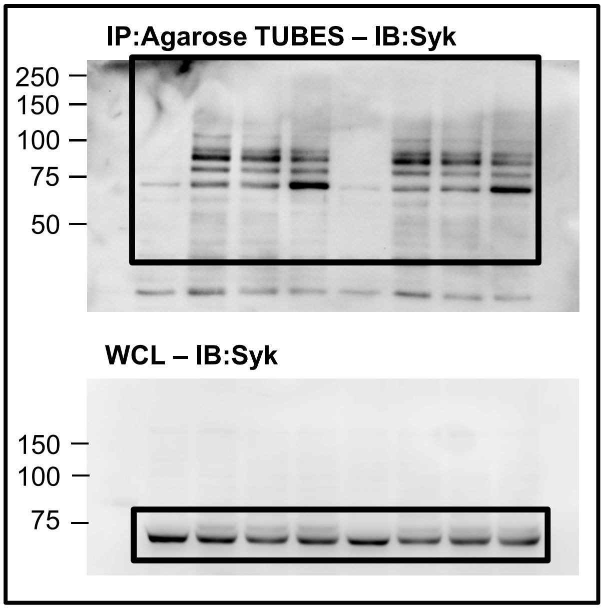

Supplementary Figure 9.

Supplementary Figure 1a

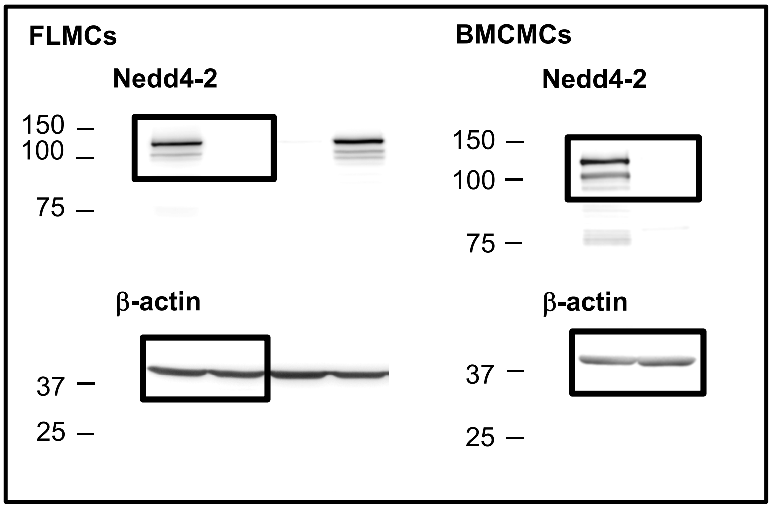

Supplementary Figure 1e

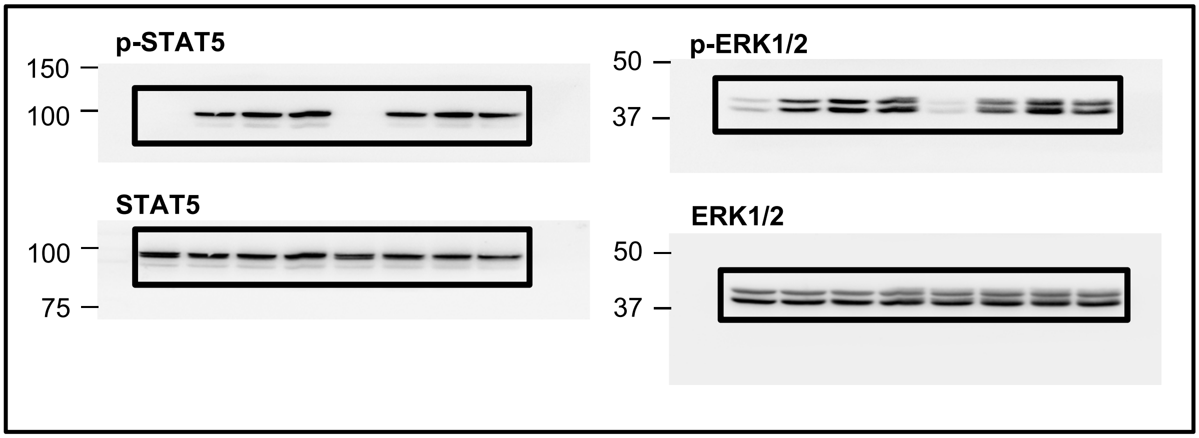

Supplementary Figure 3d

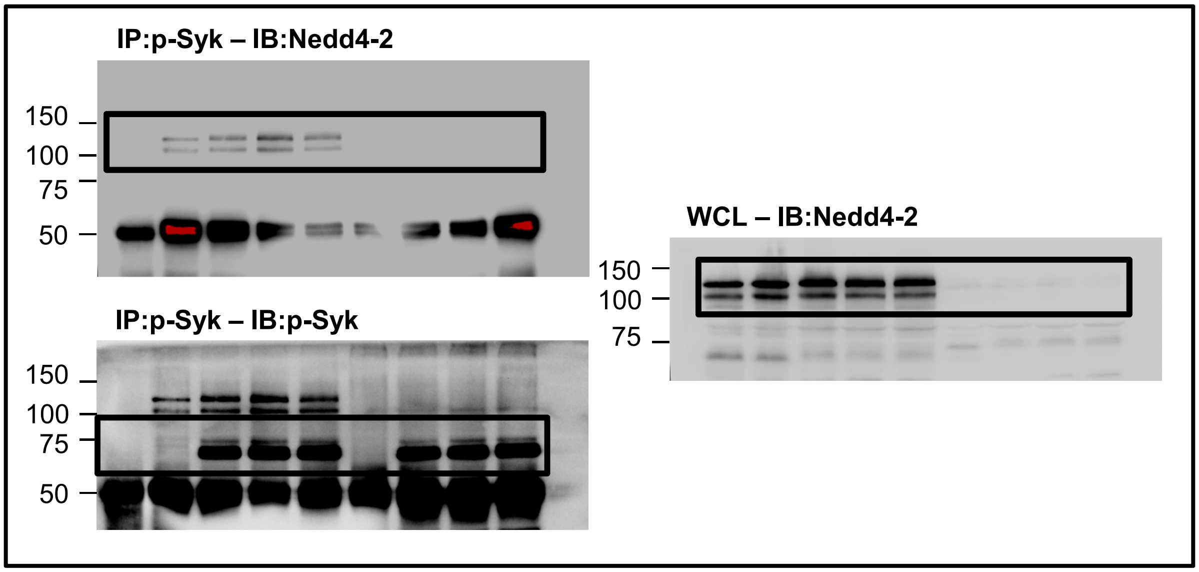

Supplementary Figure 9.

Supplementary Figure 7a

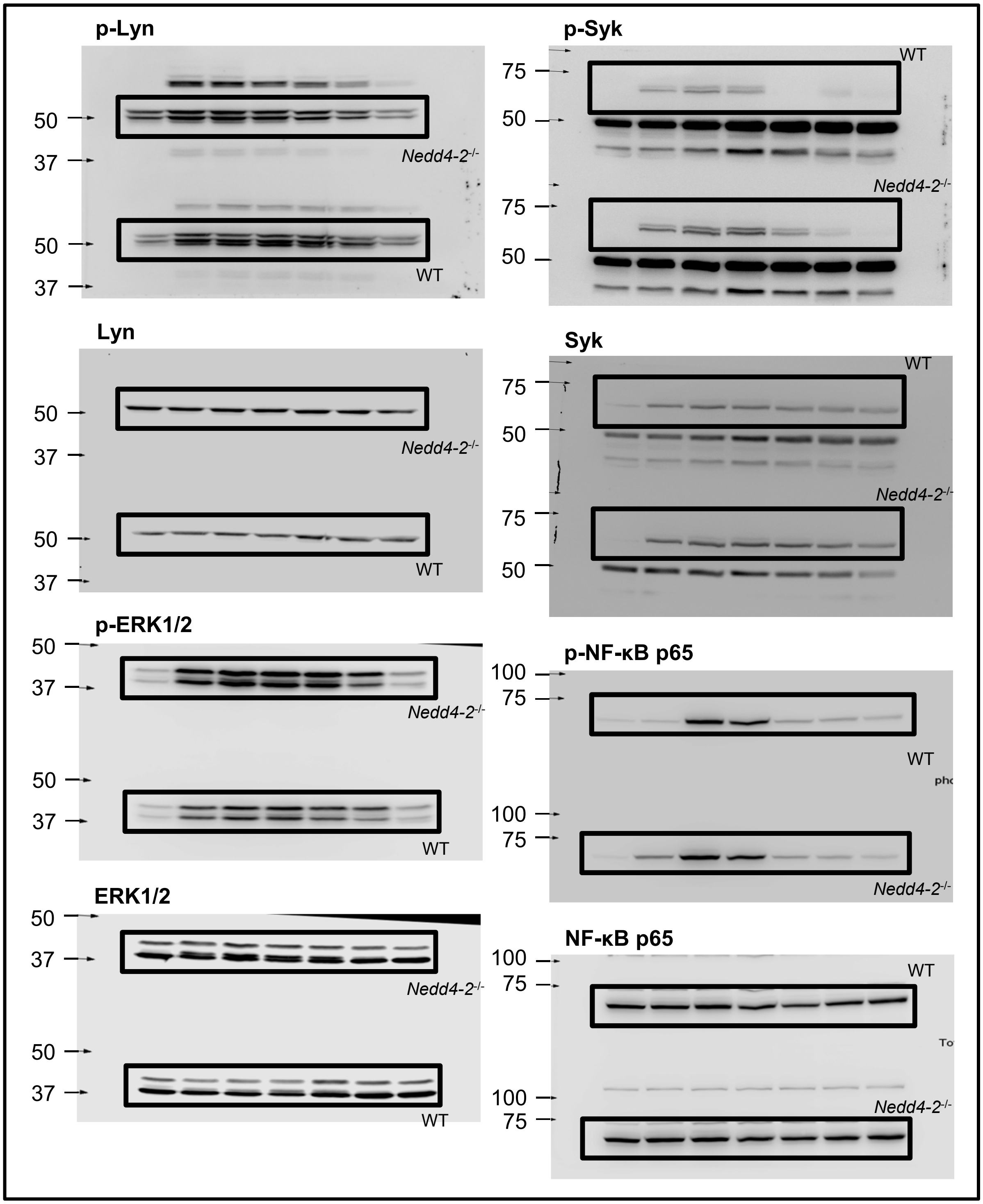

Supplementary Figure 7a

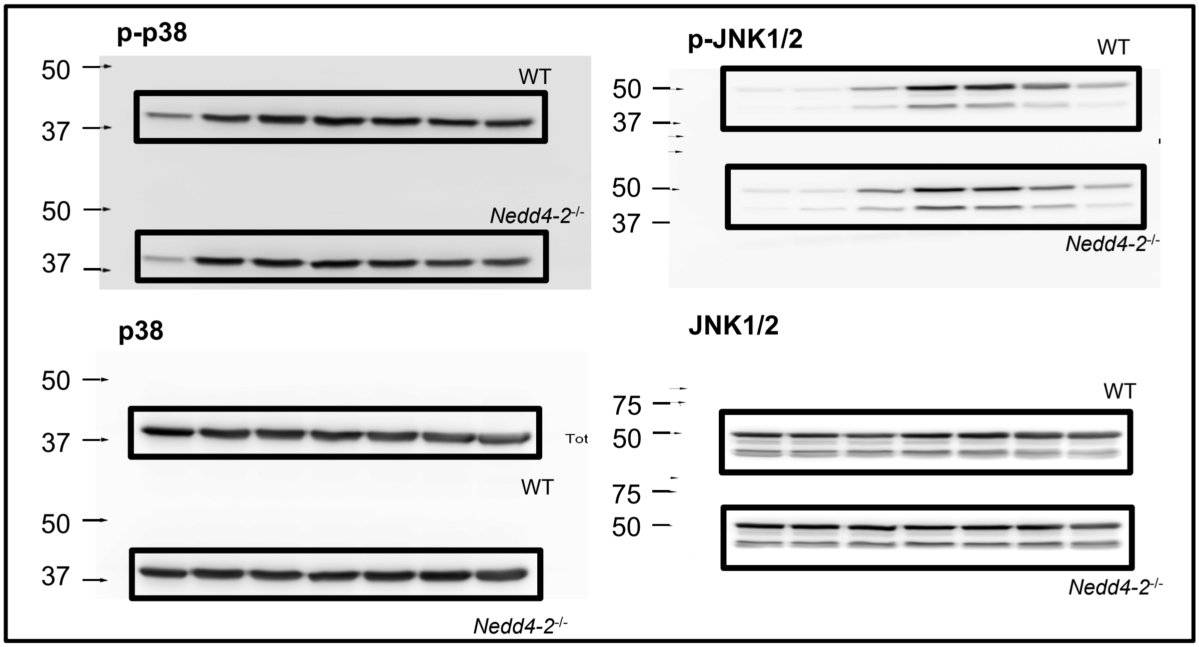

Supplementary Figure 8

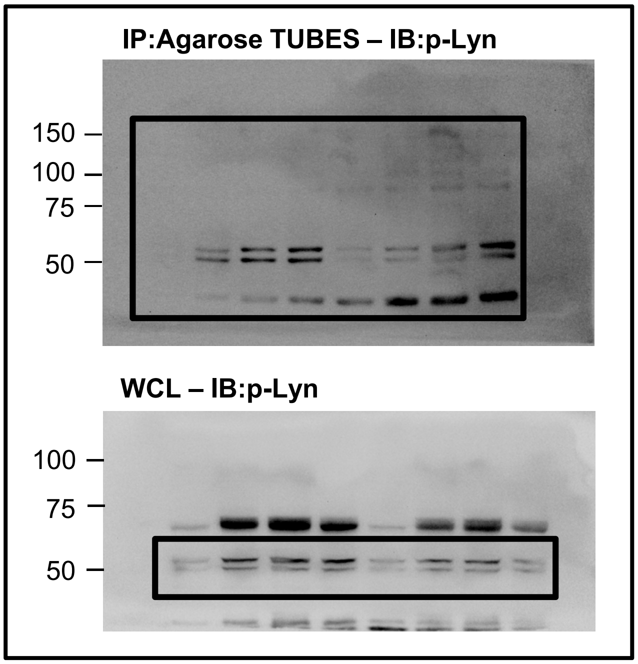

Supplementary Figure 9. Original images of immunoblots

The original immunoblots are shown with the boxed regions used in indicated figures. Molecular mass markers are indicated in kDa. For original immunoblots in supplementary Figure 7a, protein samples from two acrylamide gels were transferred onto the same membrane for detection of various proteins to ensure identical immunoblotting conditions for WT and *Nedd4-2*$^{-/-}$ samples. IP, Immunoprecipitation; IB, Immunoblot
